# Prognostic model of HIV-associated talaromycosis in south China: A large-scale retrospective study

**DOI:** 10.1371/journal.pntd.0013672

**Published:** 2025-10-30

**Authors:** Weiyin Lin, Yaozu He, Xin Chen, Mou Zeng, Huihua Zhang, Pengle Guo, Feilong Xu, Bo Liu, Xiejie Chen, Haolan He, Xiaoping Tang, Linghua Li

**Affiliations:** 1 Infectious Disease Center, Guangzhou Eighth People’s Hospital, Guangzhou Medical University, Guangzhou, Guangdong, China; 2 Guangzhou Medical Research Institute of Infectious Diseases, Guangzhou, Guangdong, China; 3 Institute of Infectious Disease, Guangzhou Eighth People’s Hospital, Guangzhou Medical University, Guangzhou, Guangdong, China; Albert Einstein College of Medicine, UNITED STATES OF AMERICA

## Abstract

**Background:**

HIV-associated talaromycosis causes substantial mortality despite available therapies. Early identification of high-risk patients remains challenging, particularly in resource-limited settings. We aimed to develop and validate a dynamic prognostic model for rapid risk stratification.

**Methods:**

This retrospective cohort study analyzed 1,892 HIV-talaromycosis patients admitted to Guangzhou Eighth People’s Hospital (2011–2023). Poor outcome (in-hospital death or deterioration-related discharge) was the primary endpoint. A nomogram was developed using Cox regression on admission variables in a training set (2011–2020, N = 1,435), with internal validation set (2011–2020, N = 431) and independent testing set (2021–2023, N = 457). Performance was assessed via time-dependent AUC, C-index, calibration, and decision curve analysis.

**Results:**

Poor outcomes occurred in 14.1% of cases (266/1,892), with 86.5% of these events happening within 28 days. Winter admissions exhibited the lowest case volume but the highest poor outcome rate. Multivariable analysis revealed eight independent readily available predictors: absence of lymphadenopathy (aHR: 0.581, 95%CI: 0.396-0.852, *P* = 0.005) and hepatosplenomegaly (aHR: 0.347, 95%CI: 0.232-0.519, *P* < 0.001), respiratory rate (aHR: 1.041, 95%CI: 1.007-1.076, *P* = 0.016), white blood cell count (aHR: 1.089, 95%CI: 1.049-1.132, *P* < 0.001), platelet count (aHR: 0.995, 95%CI: 0.992-0.997, *P* < 0.001), albumin level (aHR: 0.911, 95%CI: 0.872-0.952, *P* < 0.001), lactate dehydrogenase (aHR: 1.000, 95%CI: 1.000-1.000, *P* < 0.001), and blood urea nitrogen (aHR: 1.087, 95%CI: 1.068-1.106, *P* < 0.001). The above indicators were stratified according to predefined classifications and used to established a nomogram. The nomogram demonstrated strong discriminatory performance for 7-, 14-, and 28-day outcomes (AUC 0.905/0.863/0.838 in development; 0.851/0.832/0.807 in independent testing; C-index 0.813-0.841). Calibration curve analysis demonstrated that the nomogram exhibited excellent predictive accuracy and decision curve analysis indicated substantial clinical benefit. The model could effectively differentiate between high-risk and low-risk populations.

**Conclusion:**

This study provides a dynamically validated prognostic tool for HIV-associated talaromycosis, enabling risk stratification using readily available clinical data. Its integration into electronic health systems could off an opportunity to optimize resource allocation and improve outcomes in endemic regions.

## Author summary

HIV-associated talaromycosis continues to cause significant mortality despite current treatment options. Identifying high-risk patients early remains difficult, especially in resource-constrained settings. While prior research has identified certain prognostic factors, these studies typically involved limited sample sizes and relied solely on internal validation without external verification. Existing models also fail to evaluate mortality risk at varying time points, restricting their clinical utility. Our study examined in-hospital prognosis in HIV-associated talaromycosis patients, identifying absence of lymphadenopathy and hepatosplenomegaly, elevated respiratory rate, and abnormal white blood cell count, platelet count, albumin level, lactate dehydrogenase, and blood urea nitrogen as independent predictors of poor outcomes. Using these routinely measured clinical parameters, we developed a dynamic prediction model for risk stratification. Both internal and independent testing showed robust discrimination, high accuracy, and meaningful clinical applicability. This validated prognostic tool could effectively distinguish between high- and low-risk patients, hoping to offer a practical support for clinical decision-making and resource prioritization in endemic areas.

## Background

Disseminated talaromycosis is a systemic infection caused by Talaromyces marneffei, characterized by its invasion of the human body and dissemination through the mononuclear-macrophage system, affecting multiple organs, including the skin, lungs, liver, spleen, lymph nodes, and bone marrow [1]. This disease is predominantly endemic to southeast Asia, south China and northeast India. However, increasing number of talaromycosis cases were reported in nonendemic areas, especially in human immunodeficiency virus (HIV) infected patients with a low CD4 + T cell counts [2,3].

Talaromycosis has long been recognized as one of the most prevalent and deadly opportunistic infections in people living with advanced HIV disease. In China, Vietnam, Thailand, and India, the prevalence of talaromycosis among individuals with HIV varies between 0.1% and 26.7%, while mortality rates range from 8% to 40% [4]. Despite the introduction of antiretroviral therapy (ART) and the broad availability of antifungal agents such as amphotericin B (AMB), itraconazole, and voriconazole, the mortality rate among patients infected with TSM remains at 11.3% to 20.0% [5–9]. The high mortality rate is often attributed to delays in the identification and treatment of critically ill patients. This issue is particularly acute in primary healthcare settings, where inadequate risk assessment tools often lead to misdiagnosis or treatment delays, thereby facilitating disease progression. Consequently, there is an urgent need to develop a risk assessment model specifically for HIV-associated talaromycosis. This model should integrate fundamental clinical data, including vital signs, symptoms, and key laboratory results, to enable rapid (for example within two hours of admission) risk evaluation. Such a model would assist clinicians in rapid identification of critically ill patients, making precise medical decisions, and ultimately improving patient outcomes.

Previously we identified leucocytosis, thrombocytopenia, elevated aspartate aminotransferase (AST), total bilirubin (TBIL), creatinine (Cr), and azole monotherapy as independent predictors of higher mortality in HIV-associated talaromycosis [7]. Subsequent studies found factors such as age, septic shock, respiratory failure, pleural effusion, international normalized ratio, procalcitonin, C-reactive protein and AST/platelet (PLT) ratio index are also associated with mortality and construct predictive models for mortality in this population [2,10–14]. However, these studies generally suffer from small sample sizes, and the models have been validated only with internal test datasets, lacking independent testing, and are unable to assess mortality risk across different time intervals, thereby limiting ability to fully address the current challenges.

In this study, we aimed to investigate the prognosis during hospitalization among patients with HIV-associated talaromycosis, and construct a dynamic predictive model for risk stratification based on readily available clinical data.

## Methods

### Ethics statement

This study received approval from the Ethics Committee of Guangzhou Eighth People’s Hospital, Guangzhou Medical University (approval no. 201908121), and all enrolled patients signed the informed consent.

#### Study design and participants.

This retrospective study was conducted at Guangzhou Eighth People’s Hospital (GZ8H). We screened all patients with HIV-associated talaromycosis who admitted to GZ8H from January 1, 2011, to December 31, 2023. The inclusion criteria were: (1) age ≥ 18 years, (2) confirmed HIV infection through HIV antibody or HIV nucleic acid detection, and (3) a culture- or histopathologically confirmed diagnosis of talaromycosis. Patients lacking essential clinical data such as therapy information were excluded from the analysis. Informed consent was obtained from all enrolled patients.

#### Diagnosis of talaromycosis.

Talaromycosis was diagnosed when Talaromyces marneffei was detected through histopathology or isolated from samples like blood, bone marrow, or body fluids, which has been reported elsewhere [7,15]. The fungus was cultured on Sabouraud dextrose agar, growing as a mould at 25°C and as a yeast at 37°C. Identification relied on colony morphology, mould-to-yeast conversion, red pigment production at 25°C, and microscopic features. Histopathological diagnosis involved identifying characteristic yeast cells with a midline septum in tissue sections from lymph nodes, skin, lungs, liver, or bone marrow.

#### Data collection.

The data were sourced from the scientific research platform database of Guangzhou Eighth People’s Hospital, which containing the hospital’s electronic medical records and microbiology records. To prevent duplication, all patient entries were cross-verified in the database using identifiers such as name, date of birth, and address. The dataset encompassed demographic information, antiretroviral therapy history, clinical symptoms and signs, laboratory results including blood routine test, hepatic and renal function indexes, CD4 + T-cell count and CD8 + T-cell count ratio, antifungal therapy regimens, and outcomes during hospitalization. Tachypnea was defined as respiratory rate ≥22/min. Leucocytosis was defined as a white blood cell (WBC) count of >9.5 × 10^9^ cells/L; leucopenia, WBC count <3.5 × 10^9^ cells/L. The lower limit of normal (LLN) for hemoglobin (Hb) was 130 g/L for male and 120 g/L for female. Mild anemia was defined as a Hb between 90 g/L to LLN, moderate, Hb between 60 g/L to 89 g/L, severe, Hb < 60 g/L. The LLN for platelet was 100 × 10^9^ cells/L. Platelet was categorized as normal (>100 × 10⁹/L), 30–100 × 10⁹/L, < 30 × 10⁹/L to distinguish bleeding risk. The upper limit of normal (ULN) for total bilirubin (TBIL), alanine aminotransferase (ALT), AST, lactate dehydrogenase (LDH), alkaline phosphatase (AKP), serum Cr, blood urea nitrogen (BUN) and blood uric acid (UA) was 26 μmol/L, 50 U/L, 40 U/L, 250 U/L, 125 U/L, 111 μmol/L, 9.5 mmol/L and 428 μmol/L, respectively. Elevated TBIL, Cr, BUN, and UA levels were defined as those exceeding the ULN. ALT, AST, LDH, and AKP were categorized into three groups: < 1 × ULN, 1–5 × ULN, and >5 × ULN, to differentiate the extent of organ dysfunction and disease severity. Severe hypoalbuminemia was defined as a serum albumin (ALB) level <25 g/L.

#### Outcome measures.

Outcomes during hospitalization were categorized as follows: (1) a good outcome, defined as either cure or improvement, characterized by negative repeated blood cultures, normalization of temperature, and the disappearance or reduction of skin lesions; (2) a poor outcome, defined as in-hospital death, death at home, or clinical deterioration with an expectation of death at home (i.e., patients who were moribund or clinically deteriorated and were taken from the hospital to die at home in accordance with local customs) [7]. All outcomes were assessed by two physician investigators, with a third physician consulted in cases of uncertainty.

#### Model construction, validation and evaluation.

**Fig 1:**
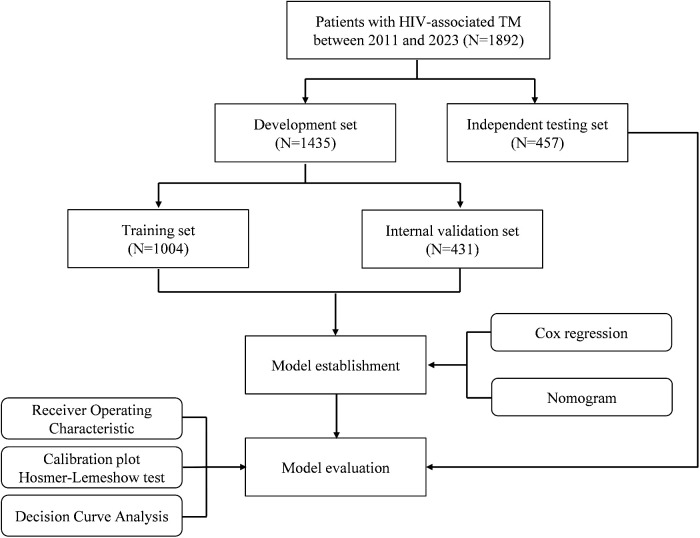
Study design and modeling workflow. Development set (2011-2020, N = 1,435) randomly split into training (N = 1,004) and internal validation (N = 431) sets; Independent testing set (2021-2023, N = 457). Cox regression-derived nomogram evaluated by receiver operating characteristic curves, calibration plots, and decision curve analysis.

This study enrolled 1,892 HIV-associated talaromycosis patients. Firstly, we used 1,435 HIV-associated talaromycosis patients admitted between 2011 and 2020 as the development set, randomly divided into a training set (N = 1004) and an internal validation set (N = 431) at a ratio of 7:3. Additionally, 457 patients admitted between 2021 and 2023 served as the independent testing set (Fig 1). We employed univariate and multivariate Cox proportional hazards regression models to identify predictors of poor outcome. To facilitate clinical application, all continuous predictors were stratified into clinically meaningful categories. We calculated each patient’s risk score of poor outcomes by linearly combining the selected stratified predictive variables with their respective coefficients. Subsequently, we developed a risk prediction nomogram using the “rms” package. The model’s performance was evaluated through discrimination and calibration analyses. Discrimination was assessed using the area under the Receiver Operating Characteristic (ROC) curve, while calibration was evaluated using calibration plots. The predictive accuracy of the risk model was evaluated by employing the C-statistic to assess discrimination and the Hosmer–Lemeshow chi-square test to evaluate calibration. Model stability was also tested by 500 rounds of bootstrap. To assess the clinical utility of our model, we conducted Decision Curve Analysis (DCA), which calculates the net benefits of the model across various threshold probabilities, balancing true positives and false positives [16]. DCA facilitates the identification of clinically relevant threshold ranges where our model offers significant decision-making advantages. The procedures for model development, along with the assessment of discrimination and calibration performance, were executed using comparable methodologies. Finally, we determined the optimal cutoff value for the nomogram scores using the surv_cutpoint function from the R package survminer, stratifying patients into high-risk and low-risk groups. Prognostic differences between these groups were then assessed through Kaplan-Meier survival analysis with log-rank tests.

#### Statistical analysis.

The majority of laboratory indicators, such as WBC, Hb, PLT, TBIL, ALT, AST, ALB, LDH, AKP, BUN, UA and Cr, were classified according to established normal reference ranges. Categorical variables were represented as numbers and percentages, whereas continuous variables were described using either the median and interquartile range (IQR) or the mean and standard deviation, contingent upon the data distribution. The assumption of normality was evaluated using the Kolmogorov–Smirnov test. To examine qualitative differences between subgroups, either the Chi-square test or Fisher’s exact test was employed. For the assessment of quantitative differences, the Student’s t-test or Mann-Whitney test was utilized. All statistical analyses were conducted using two-sided tests, with a *P*-value of less than 0.05 considered indicative of statistical significance. All statistical analyses, data visualizations and drawing of the figures were performed using R (version 4.4.2) (http://www.R-project.org), JD_PCPM (V4.31, Jingding Medical Technology Co., Ltd.) and SPSS software, version 26.0 (IBM, Armonk, NY).

## Results

### Baseline characteristics

This study enrolled 1,892 HIV-associated talaromycosis patients (median age 38.0 years; 81.6% male), with 15.1% reporting prior ART exposure (Table 1). During a median hospitalization period of 27.0 days, 266 (14.1%) patients had poor outcome, including 163 in-hospital deaths and 103 discharges with clinical deterioration (Table 1). Compared to good outcome patients, poor outcome patients were older (median 41.0 years vs 38.0 years, *P* = 0.001) with reduced incidence of lymphadenopathy (49.2% vs 72.1%, *P* < 0.001) and hepatosplenomegaly (42.1% vs 59.8%, *P* < 0.001), but higher digestive involvement (49.2% vs 37.7%, *P* < 0.001). Hemodynamic instability was more prominent featuring by elevated breath rate (21/min vs 20/min) and shock index (1.0 vs 0.9, all *P* < 0.05). Hematological abnormalities included leukocytosis (19.2% vs 5.8%), severe thrombocytopenia (56.5 × 10⁹/L vs 129.0 × 10⁹/L), and severe anemia (13.9% vs 6.9%, all *P* < 0.001) were more prevalent. In addition, multi-organ dysfunction dominated in poor prognosis cases: hepatic injury (bilirubin 17.5 μmol/L vs 9.3 μmol/L, AST > 5 × ULN in 45.4% vs 14.0%), severe hypoalbuminemia (<25 g/L in 70.3% vs 39.4%), and renal impairment (BUN elevation in 42. %vs 6.8%; creatinine elevation in 34.6% vs 7.4%, all *P* < 0.05). Systemic inflammation markers surged (LDH 870.0 U/L vs 405.5 U/L, *P* < 0.001), accompanied by CD8 + T-cell depletion (185.5/μL vs 238.0/μL, *P* < 0.001).

**Table 1:** Baseline characteristics of study participants.

Characters	Total (N = 1892)	Outcomes		
		Good (N = 1626)	Poor (N = 266)	*P*-value
Age (yes)	38.0 [30.0;48.0]	38.0 [30.0;47.0]	41.0 [32.0;50.0]	0.001
Male, n (%)	1544 (81.6%)	1337 (82.2%)	207 (77.8%)	0.102
ART experienced, n (%)	285 (15.1%)	246 (15.1%)	39 (14.7%)	0.916
Fever, n (%)	1449 (76.6%)	1234 (75.9%)	215 (80.8%)	0.092
Respiratory systems, n (%)	1262 (66.7%)	1090 (67.0%)	172 (64.7%)	0.489
Digestive systems, n (%)	744 (39.3%)	613 (37.7%)	131 (49.2%)	<0.001
Skin lesions, n (%)	810 (42.8%)	680 (41.8%)	130 (48.9%)	0.037
Lymphadenopathy, n (%)	1303 (68.9%)	1172 (72.1%)	131 (49.2%)	<0.001
Hepatosplenomegaly, n (%)	1084 (57.3%)	972 (59.8%)	112 (42.1%)	<0.001
Breath rate (/min)	20.0 [20.0;21.0]	20.0 [20.0;21.0]	21.0 [20.0;24.0]	<0.001
Shock index	0.9 [0.8;1.1]	0.9 [0.8;1.1]	1.0 [0.8;1.2]	<0.001
WBC (10^9^)	4.1 [2.8;6.0]	4.0 [2.7;5.8]	4.8 [3.0;8.0]	<0.001
WBC stratifies				<0.001
Normal	1035 (54.7%)	909 (55.9%)	126 (47.4%)	
Leucopenia	711 (37.6%)	622 (38.3%)	89 (33.5%)	
Leucocytosis	146 (7.7%)	95 (5.8%)	51 (19.2%)	
Hb (g/L)	91.4 (22.3)	92.3 (21.8)	85.8 (24.6)	<0.001
Anemia stratifies				<0.001
Normal	209 (11.0%)	185 (11.4%)	24 (9.0%)	
Mild (90 g/L -LLN)	787 (41.6%)	703 (43.2%)	84 (31.6%)	
Moderate (60 g/L -89 g/L)	747 (39.5%)	626 (38.5%)	121 (45.5%)	
Severe (<60 g/L)	149 (7.9%)	112 (6.9%)	37 (13.9%)	
PLT (10^9^)	117.5 [56.0;197.0]	129.0 [64.0;205.0]	56.5 [31.0;112.5]	<0.001
Thrombocytopenia stratifies				<0.001
Normal	1048 (55.4%)	971 (59.7%)	77 (28.9%)	
30*10^9^-100*10^9^	643 (34.0%)	517 (31.8%)	126 (47.4%)	
< 30*10^9^	201 (10.6%)	138 (8.5%)	63 (23.7%)	
TBIL (μmol/L)	9.9 [7.0;17.3]	9.3 [6.7;15.1]	17.5 [9.0;32.8]	<0.001
TBIL elevation	260 (13.7%)	177 (10.9%)	83 (31.2%)	<0.001
ALT (U/L)	35.0 [21.0;62.0]	34.0 [21.0;60.0]	45.0 [25.0;72.0]	<0.001
ALT stratifies				<0.001
< 1 ULN	1231 (65.1%)	1085 (66.7%)	146 (54.9%)	
1–5 ULN	623 (32.9%)	521 (32.0%)	102 (38.3%)	
> 5 ULN	38 (2.0%)	20 (1.2%)	18 (6.8%)	
AST (U/L)	76.0 [39.0;159.2]	69.0 [37.0;139.8]	170.0 [75.2;389.0]	<0.001
AST stratifies				<0.001
< 1 ULN	485 (25.6%)	456 (28.0%)	29 (10.9%)	
1–5 ULN	1058 (55.9%)	942 (57.9%)	116 (43.6%)	
> 5 ULN	349 (18.4%)	228 (14.0%)	121 (45.5%)	
ALB	25.0 [22.0;29.0]	26.0 [22.0;30.0]	22.0 [19.0;25.0]	<0.001
Severe hypoalbuminemia (<25 g/L)	828 (43.8%)	641 (39.4%)	187 (70.3%)	<0.001
LDH (U/L)	437.0 [295.0;776.0]	405.5 [285.0;679.8]	870.0 [435.0;1844.0]	<0.001
LDH stratifies				<0.001
< 1 ULN	285 (15.1%)	271 (16.7%)	14 (5.3%)	
1–5 ULN	1360 (71.9%)	1205 (74.1%)	155 (58.3%)	
> 5 ULN	247 (13.1%)	150 (9.2%)	97 (36.5%)	
AKP (U/L)	128.0 [82.0;237.0]	122.0 [80.0;226.8]	169.5 [102.0;296.0]	<0.001
AKP stratifies				0.001
< 1 ULN	935 (49.4%)	832 (51.2%)	103 (38.7%)	
1–5 ULN	891 (47.1%)	742 (45.6%)	149 (56.0%)	
> 5 ULN	66 (3.5%)	52 (3.2%)	14 (5.3%)	
BUN (mmol/L)	4.5 [3.3;6.3]	4.2 [3.2;5.7]	7.9 [5.0;14.2]	<0.001
BUN elevation	224 (11.8%)	110 (6.8%)	114 (42.9%)	<0.001
UA (μmol/L)	238.2 [177.5;315.0]	233.8 [176.0;303.0]	261.9 [187.2;429.8]	<0.001
UA elevation	184 (9.7%)	116 (7.1%)	68 (25.6%)	<0.001
Cr (μmol/L)	68.1 [57.0;85.0]	67.0 [56.0;82.0]	85.0 [62.1;150.8]	<0.001
Cr elevation:	212 (11.2%)	120 (7.4%)	92 (34.6%)	<0.001
CD4 (cells/μL)	11.0 [5.0;23.0]	11.5 [5.0;24.0]	11.0 [4.0;21.0]	0.163
CD8 (cells/μL)	233.0 [123.0;395.2]	238.0 [128.0;411.5]	185.5 [85.5;307.0]	<0.001
CD4/CD8 ratio	0.05 [0.03;0.09]	0.05 [0.03;0.09]	0.06 [0.03;0.10]	0.144

*P*^1^ represents the comparison between the training set and the internal verification set, and *P*^2^ represents the comparison between the training set and the independent testing set. Categorical variables were represented as n (%), whereas continuous variables were described using either the median and interquartile range (IQR) or the mean and standard deviation, contingent upon the data distribution.

ART: antiretroviral therapy; n (%): number (percentage); IQR: interquartile range; WBC: white blood cell; Hb: hemoglobin; LLN: lower limit of normal; PLT: platelet; TBIL: total bilirubin; ALT: alanine aminotransferase; ULN: upper limit of normal; AST: aspartate aminotransferase; ALB: albumin; LDH: lactate dehydrogenase; AKP: alkaline phosphatase; BUN: blood urea nitrogen; UA: uric acid; Cr: creatinine.

### Treatment and outcomes

Within the entire 1,892 study population, 57.6% (N = 1090) of patients received amphotericin B (AMB) in combination with azoles, 40.8% (N = 772) were administered azole monotherapy (such as voriconazole, itraconazole, or fluconazole), and 1.6% (N = 30) did not receive any antifungal therapy (S1A Fig). Information regarding antiretroviral therapy (ART) was available for 813 patients who admitted between January 1, 2018 and December 31, 2023. The analysis indicated that 60.4% (N = 491) were prescribed regimens consisting of NRTIs and INSTIs, 7.9% (N = 64) received NRTIs in combination with NNRTIs, 3.8% (N = 31) were treated with NRTIs and PIs, 2.5% (N = 20) initially received only NRTIs but added a third class of drugs post-discharge, and 25.5% (N = 207) commenced ART within 2 weeks after discharge (S1B Fig).

The annual rates of poor outcome demonstrated variability, with a minimum of 9.0% (8/89) in 2011 and a maximum of 25.2% (27/107) in 2021 (*P* = 0.010). Despite these interannual fluctuations, the data did not exhibit a consistent linear trend over time, either increasing or decreasing (S1C Fig). A seasonal analysis revealed a significant pattern (**P* *= 0.034) (S1D Fig). Winter, which had the lowest absolute number of cases (N = 360) compared to other seasons—less than half the number of cases in spring (N = 681) and significantly fewer than in summer (N = 503)—displayed a disproportionately high rate of poor outcomes at 18.3% (66 out of 360). This rate surpassed that of summer (12.3%, *P* = 0.014) and autumn (11.5%, *P* = 0.011).

### Development and validation of a prognostic model

The analysis revealed that 86.5% (230/266) of the poor outcome occurred within 28 days of admission. The cumulative incidence rates of poor outcomes at 7, 14, and 28 days were 8.2%, 10.4%, and 13.4%, respectively (S2 Fig). Thus, we construct a prognostic model based on essential clinical data at early admission to predict occurrence of poor outcome within 28 days. The training and internal validation sets demonstrated comparable baseline characteristics across all parameters, except for serum creatinine (S1 Table). In contrast, the independent testing set diverged significantly from the training set in age distribution, male predominance, ART experience, and clinical manifestations. Besides, laboratory disparities involved higher CD4 + T-cell counts, lower LDH, and reduced hypoalbuminemia. However, vital signs, hemoglobin, platelet counts, and renal markers showed no significant differences between training and independent testing sets.

**Fig 2:**
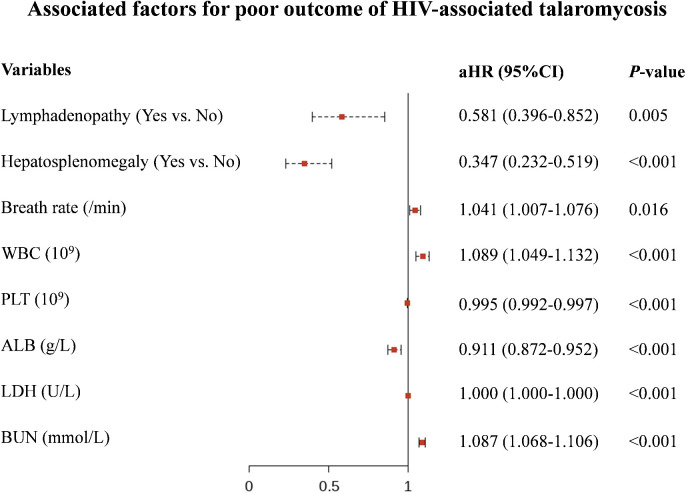
Independent predictors of poor outcome. Forest plot showing adjusted hazard ratios (aHR) and 95% confidence interval (95% CI): absence of lymphadenopathy (aHR 0.581, 95% CI 0.396-0.852), absence of hepatosplenomegaly (aHR 0.347, 95% CI 0.232-0.519), elevated breath rate (per breath/min increase: aHR 1.041, 95% CI 1.007-1.076), increased white blood cell count (per 1 × 10⁹/L increase: aHR 1.089, 95% CI 1.049-1.132), decreased platelet count (per 1 × 10⁹/L decrease: aHR 0.995, 95% CI 0.992-0.997), hypoalbuminemia (per 1 g/L decrease: aHR 0.911, 95% CI 0.872-0.952), elevated lactate dehydrogenase (per 1 U/L increase: aHR 1.000, 95% CI 1.000-1.000), and increased blood urea nitrogen (per 1 mmol/L increase: aHR 1.087, 95% CI 1.068-1.106). All P < 0.05. For clinical application, these continuous variables will be categorized before developing the nomogram.

**Fig 3:**
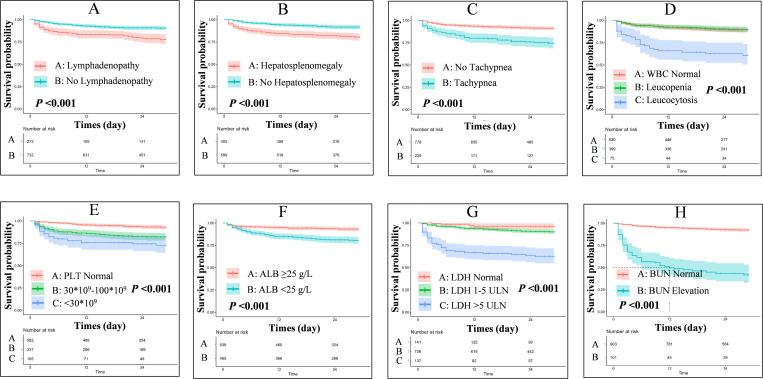
Survival analysis by predictor stratification. (A-H) Significant differences in poor outcome risk across stratified groups of lymphadenopathy, hepatosplenomegaly, tachypnea, white blood cell count, platelet count, albumin, lactate dehydrogenase, and blood urea nitrogen (all log-rank P < 0.001).

**Fig 4:**
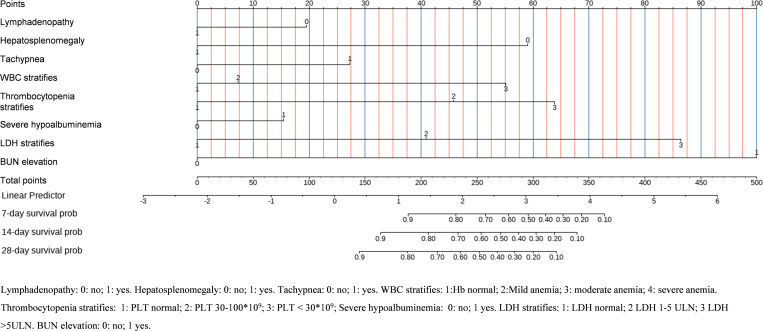
Dynamic nomogram for risk prediction. Clinical tool integrating eight stratified predictors to estimate 7-day/14-day/28-day poor outcome probabilities.

We identify predictors for poor outcome through univariate and multivariate Cox regression analysis in the training set. We found that absence of lymphadenopathy (aHR: 0.581, 95%CI: 0.396-0.852, *P* = 0.005), absence of hepatosplenomegaly (aHR: 0.347, 95%CI: 0.232-0.519, *P* < 0.001), increased breath rate (aHR: 1.041, 95%CI: 1.007-1.076, *P* = 0.016), higher WBC count (aHR: 1.089, 95%CI: 1.049-1.132, *P* < 0.001), lower PLT count (aHR: 0.995, 95%CI: 0.992-0.997, *P* < 0.001), lower ALB level (aHR: 0.911, 95%CI: 0.872-0.952, *P* < 0.001), higher LDH level (aHR: 1.000, 95%CI: 1.000-1.000, *P* < 0.001), and higher BUN level (aHR: 1.087, 95%CI: 1.068-1.106, *P* < 0.001) were independently associated with higher risk of poor outcome (Fig 2 and S2 Table). To facilitate clinical application, we stratified the continuous indicators according to established classifications and tested their clinical meaning. Survival analysis indicated that these stratified indicators can differentiate between various risk levels (Fig 3). Based on the stratified indicators, we constructed a nomogram model to predict the 7-day, 14-day and 28-day probabilities of poor outcome among HIV-associated talaromycosis (Fig 4).

**Fig 5:**
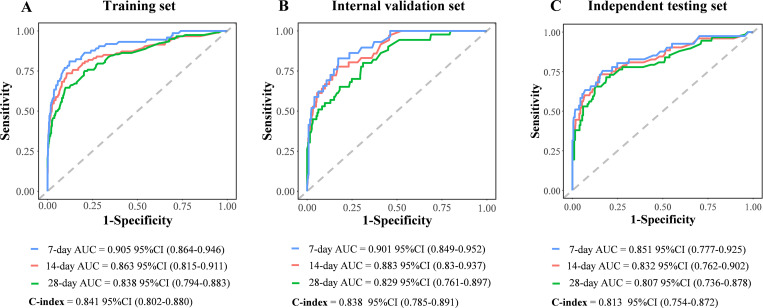
Receiver operating characteristic curve analysis of the prognostic model. (A) Training set: 7-day area under the curve (95% confidence interval) = 0.905 (0.864-0.946), 14-day = 0.863 (0.815-0.911), 28-day = 0.838 (0.794-0.883); Concordance index = 0.841 (0.802-0.880). (B) Internal validation set: 7-day area under the curve = 0.901 (0.849-0.952), 14-day = 0.883 (0.830-0.937), 28-day = 0.829 (0.761-0.897); Concordance index = 0.838 (0.785-0.891). (C) Independent testing set: 7-day area under the curve = 0.851 (0.777-0.925), 14-day = 0.832 (0.762-0.902), 28-day = 0.807 (0.736-0.878); Concordance index = 0.813 (0.754-0.872).

**Fig 6:**
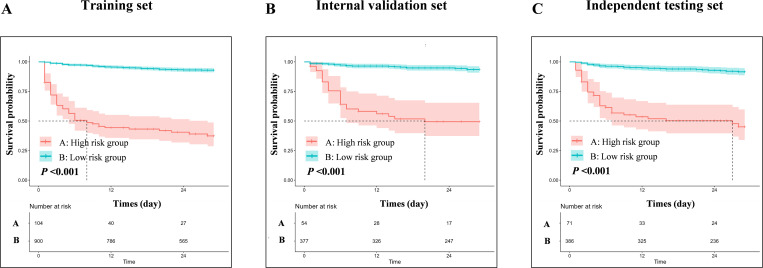
Risk stratification survival analysis. (A-C) Significant difference in survival probability between high-risk and low-risk groups defined by nomogram scores in (A) training, (B) internal validation, and (C) independent testing sets (all log-rank P < 0.001).

The evaluations of the nomogram model were conducted through internal and independent testings. First, the discriminative ability of the model to differentiate between good outcome and poor outcome groups was evaluated using the receiver operating characteristic (ROC) curve and concordance index (C-index). Specifically, the area under the ROC for 7-day, 14-day, and 28-day predictions was high, reaching 0.905, 0.863, and 0.838 in the development set, 0.901, 0.883, and 0.829 in the internal validation set, and 0.851, 0.832, and 0.807 in the independent testing set, respectively (Fig 5). The C-index of the nomogram model in the development set, internal validation set and independent testing set was 0.841, 0.838, and 0.813, respectively. In addition, time-dependent AUC curves and time-dependent C-index curves demonstrated robust stability of these parameters across different time points (S3 Fig). Subsequently, the agreement between the predicted probabilities and the observed probabilities was assessed. Calibration curve analysis demonstrated that the nomogram model exhibited excellent predictive accuracy in all sets, with predicted risks closely matching observed outcomes at clinically critical time points (S4 Fig). Finally, the net benefit (NB) of the nomogram model in clinical decision-making was evaluated, balancing the benefits of true-positive predictions against the costs of false-positive results. Decision curve analysis (DCA) results indicated that the nomogram model established in this study provided substantial clinical benefit (S5 Fig). In the training set, internal validation set, and independent test set, patients with HIV-associated talaromycosis in the low-risk group showed a 28-day survival rate above 90%, whereas the high-risk group exhibited a rate below 50%. Risk stratification analysis revealed that the nomogram model could effectively differentiate between high-risk and low-risk populations (Fig 6).

## Discussion

This large-scale retrospective study established and validated a novel prognostic model for predicting poor outcome including in-hospital mortality and clinical deterioration in HIV-associated talaromycosis. By analyzing 1,892 patients over 13 years, we identified eight readily available clinical indicators that synergistically reflect the pathophysiological triad of immune dysregulation, systemic inflammation, and end-organ damage. The resulting dynamic nomogram demonstrated exceptional clinical utility for risk stratification throughout the critical 28-day hospitalization period.

It is not surprised to found that the absence of lymphadenopathy and hepatosplenomegaly, along with tachypnea, leukocytosis, thrombocytopenia, severe hypoalbuminemia, elevated LDH, and increased BUN, predicted poor outcomes in HIV-associated talaromycosis. These markers define a high-risk phenotype characterized by immune exhaustion, systemic inflammation, and organ dysfunction. Our previous study reported that splenomegaly and hepatomegaly occurred less frequently in patients with poor outcome than in those with good outcome [7]. The absence of lymphadenopathy or hepatosplenomegaly during Talaromyces marneffei infection may indicate a compromised ability of the mononuclear phagocyte system (MPS) to contain the pathogen. Typically, the MPS sequesters pathogens within lymphoid tissues, resulting in lymphadenopathy or hepatosplenomegaly as part of the immune response. Mononuclear phagocytes which express CD4 receptor and chemokine co-receptors represent important cellular targets for HIV-1. The envelope glycoprotein specifically compromises the functionality of mononuclear phagocytes by impairing their maturation and cytokine secretion [17]. When HIV infects monocytes, it may impair the MPS’s capacity for pathogen containment. Consequently, the pathogen may disseminate more extensively, leading to a severe systemic infection without the typical manifestations of a localized immune response. Besides, Talaromyces marneffei could also manipulate host immunity to enhance survival and replication. Shen L et la found that Talaromyces marneffei alters macrophage polarization via arginine metabolism, promoting an M2 phenotype with reduced antimicrobial activity [18]. Fang J et al reported that this fungus can capture CD86 from macrophages, potentially disrupting co-stimulatory signals required for T-cell activation [19]. Other predictors—tachypnea, leukocytosis, thrombocytopenia, hypoalbuminemia, elevated LDH, and increased BUN—have been consistently linked to poor outcomes in previous studies [12–14]. Leukocytosis and thrombocytopenia may reflect proliferation of Talaromyces marneffei in macrophages followed by systemic dissemination and bone marrow infiltration [20]. Severe hypoalbuminemia suggests hepatic dysfunction secondary to sepsis, while tachypnea, elevated LDH, and increased BUN indicate hypoxia, cellular lysis, renal hypoperfusion, and microcirculatory disturbances [1,21]. This specific combination of markers precisely captures the pathophysiology of Talaromyces marneffei infection. In the context of cellular immunodeficiency, Talaromyces marneffei employs intracellular parasitism and immune evasion to invade and proliferate within the mononuclear phagocyte system, leading to silent immune collapse and disseminated multi-organ damage. Although unit change (U/L) of LDH contributes little to risk stratification when used as a continuous variable (aHR: 1.000, 95%CI: 1.000-1.000, P < 0.001), when used as a categorical variable, it can be found that the cumulative incidence of adverse outcomes exceeds 30% in patients with LDH > 5 ULN (Fig 3G). Together, these markers delineate a pathological cascade of Talaromyces marneffei dissemination, immune collapse, microcirculatory dysfunction, and multi-organ failure.

Previously Shi et al developed an XGBoost model predicting in-hospital mortality in 1927 HIV/AIDS patients with Talaromyces marneffei using 15 admission features, identifying septic shock and platelet count as top predictors [10]. Despite strong internal validation, their model lacked temporal risk stratification and independent testing, and did not incorporate pathophysiological indicators like immune exhaustion markers. The present large-scale study containing 1892 patients with HIV-associated talaromycosis, employed Cox proportional hazards regression incorporating temporal dynamics to achieve stratified risk prediction at 7/14/28 days (AUC 0.83-0.91), thereby better aligning with clinical decision-making time windows. Notably, this study innovatively integrated immune exhaustion markers, specifically the absence of lymphadenopathy and hepatosplenomegaly, highlighting MPS dysregulation as the central pathological mechanism underlying Talaromyces marneffei dissemination, providing an immunological basis for prognostic evaluation. In addition, the present study focuses on eight readily available clinical indicators, offering greater practicality than previous study requiring fifteen parameters. This streamlined approach facilitates implementation in primary healthcare settings. Finally, in terms of model validation, this study transcended the limitations of single-center internal validation by incorporating an independent testing set (N = 457, 2021–2023 data) with distinct clinical characteristics, confirming the model’s cross-population stability (C-index 0.81-0.84) and further enhancing its generalizability in clinical practice.

The clinical translational value of this predictive model can be demonstrated by its capacity to facilitate prediction-based precision interventions. Specifically, the risk score calculated using data obtained within a short admission period enables rapid identification of patients with >50% predicted 28-day mortality risk. By implementing this model, clinicians can efficiently screen high-risk patients, thereby providing a reliable basis for subsequent precision interventions. These interventions may include timely initiation of amphotericin B therapy, referral from primary to tertiary care hospitals, or prompt ICU admission for intensive care management.

In addition to the above eight modeling parameters, significant seasonal variations were observed both in the incidence of HIV-associated Talaromyces marneffei infections and poor outcome rates. This phenomenon can be attributed to the abundant rainfall during spring and summer, which creates a humid climate conducive to the growth of Talaromyces marneffei, thereby increasing the susceptibility of immunocompromised individuals to talaromycosis. Surprisingly, winter exhibited the lowest incidence rate but the highest mortality (18.3%), significantly surpassing those of summer (12.3%) and autumn (11.5%). Clinically, patients diagnosed in winter presented with a lower prevalence of fever and lymphadenopathy upon admission but demonstrated higher rates of dyspnea, severe hypoalbuminemia, and elevated BUN, suggesting more advanced immune exhaustion and organ failure at the time of hospitalization, which needs more studies to investigate the mechanisms in the future (S3 Table). The antifungal treatment regimen was identified as a critical prognostic factor. Our previous research demonstrated that azole monotherapy was associated with a higher incidence of adverse outcomes compared to regimens containing amphotericin B, and azole monotherapy was independently correlated with poor clinical outcomes [7]. The present study revealed that the poor outcome rate in patients receiving azole monotherapy (18.7%, 144/772) was significantly higher than in those treated with amphotericin B-based regimens (10.3%, 112/1090, *P* < 0.001) (S6 Fig). Multivariate COX regression analysis identified AMB (aHR: 0.040, 95%CI: 0.272-0.599, *P* < 0.001) as an additional independent predictor of adverse outcomes (S4 Table), which was consistent with our prior findings.

This study has several limitations. First, the retrospective design and reliance on readily available admission data from primary care settings may overlook longitudinal factors affecting outcomes, including fluctuations in antifungal treatments, ART adjustments, or inflammatory marker trends, as well as undocumented variables like pathogen- or immunity-specific indicators (e.g., Mp1p antigen, CD86 capture), pathogen burden or genetic susceptibility. Second, although temporal validation with 2021–2023 admissions supports external reproducibility, the single-center data source requires multicenter verification to confirm applicability across varied populations and clinical settings. Third, despite strong discriminatory performance (AUC 0.83 - 0.91, C-index 0.81-0.84) in our analyses, prospective comparisons with conventional ICU scoring systems (e.g., SOFA, APACHE II, SAPS II) are needed to assess incremental clinical value and refine risk categorization. Finally, although the model parameters largely reflect systemic inflammation and organ dysfunction in advanced HIV infection, this study specifically enrolled patients with culture- or histopathologically confirmed T. marneffei infection, data from patients with other opportunistic infections, such as pneumocystis pneumonia, cytomegalovirus pneumonia, cryptococcal meningitis, or tuberculosis, are lacking. Therefore, this model cannot yet be extrapolated to all advanced HIV patients.

This study provides a dynamically validated prognostic tool for HIV-associated talaromycosis, enabling risk stratification using readily available clinical data. Its integration into electronic health systems could off an opportunity to optimize resource allocation and improve outcomes in endemic regions.

## Supporting information

S1 Data Development set data. (PDF)

S2 Data Internal validation set data. (PDF)

S3 Data Independent testing set data. (PDF)

S1 Table Comparation of baseline characteristics between training set, internal validation set and external validation set. (DOCX)

S2 Table Univariate and multivariate analysis of factors associated with poor outcome of HIV-associated talaromycosis. (DOCX)

S3 Table Comparation of baseline characteristics between patients admitted at winter and non-winter. (DOCX)

S4 Table Antifungal therapy-adjusted multivariate analysis. (DOCX)

S1 Fig Treatment patterns and seasonal outcomes. (A) Antifungal therapy distribution: amphotericin B (AMB)-based regimens (57.6%), azole monotherapy (40.8%), no therapy (1.6%); (B) Antiretroviral therapy distribution for 813 patients who admitted between January 1, 2018 and December 31, 2023; (C) Interannual poor outcome rates (2011–2023, *P* = 0.010); (D) Seasonal incidence and poor outcome rates (winter: 18.3% vs. summer: 12.3%, autumn: 11.5%; *P* = 0.034).
(TIF)

S2 Fig Cumulative incidence of poor outcomes. 86.5% (230/266) of poor outcomes occurred within 28 days (7-day: 8.2%, 14-day: 10.4%, 28-day: 13.4%).
(TIF)

S3 Fig Time-dependent discrimination performance of the prognostic model. (A-C) Time-dependent area under the curve values for 28-day prediction; (D-F) Concordance index stability over time in (A, D) training, (B, E) internal validation, and (C, F) independent testing sets. Gray band indicates 500 rounds of bootstrap confidence intervals.
(TIF)

S4 Fig Calibration plots assessing prediction accuracy. (A-C) Agreement between predicted and observed poor outcome probabilities at 7, 14, and 28 days in (A) training, (B) internal validation, and (C) independent testing sets. Dashed line represents perfect calibration.
(TIF)

S5 Fig Decision curve analysis evaluating clinical utility. (A-I) Net benefit of the nomogram compared to “treat all” or “treat none” strategies across threshold probabilities (0–100%) for: (A, D, G) training set; (B, E, H) internal validation; (C, F, I) independent testing.
(TIF)

S6 Fig Comparison of poor outcome rates by antifungal regimen. Bar plot demonstrating significantly lower incidence of poor outcomes in patients receiving amphotericin B-azole combination therapy (10.3%, 112/1090) compared to azole monotherapy (18.7%, 144/772) (P < 0.001).
(TIF)
