# Supplementary material for: Prognostic model of HIV-associated talaromycosis in south China: A large-scale retrospective study
Source: PLoS Negl Trop Dis. 2025 Oct 30;19(10):e0013672. doi: 10.1371/journal.pntd.0013672 (PMC12591474; doi:10.1371/journal.pntd.0013672)
Supplement: S1 Data — (PDF) [file pntd.0013672.s001.pdf]

study_ID	Poor_outc	Time_28D	Lymphade	Hepatospl	Tachypnea	WBC_strat	Thromboc	Severe_hy	LDH_strati	BUN_elevation
415	1	1	0	0	0	3	2	1	3	1
463	0	23	1	1	0	2	1	0	2	0
179	0	25	1	0	0	1	1	1	2	0
526	0	29	1	1	0	2	1	0	2	0
195	0	20	1	1	1	1	2	1	2	0
938	0	29	1	0	0	1	1	1	3	0
1163	0	29	1	1	0	2	2	1	3	0
1355	0	23	1	1	0	2	1	0	2	0
1279	0	29	1	1	0	2	2	1	2	0
1296	0	25	0	0	0	2	1	0	2	0
1038	0	26	0	1	1	2	2	1	3	0
1433	1	3	0	0	1	2	2	0	2	1
665	1	15	1	0	1	3	2	1	2	0
602	0	29	1	0	0	1	1	1	2	0
709	0	29	0	0	0	1	1	1	2	0
1011	0	29	0	1	1	1	1	0	2	1
1135	0	29	1	0	0	2	1	1	2	0
953	0	29	0	1	0	2	2	1	2	0
348	0	26	1	0	0	1	1	0	1	0
1017	0	28	0	0	0	1	1	0	2	0
1413	0	29	1	1	0	2	2	1	2	0
840	0	23	1	1	0	2	1	1	2	0
26	0	28	1	1	0	2	1	0	1	0
519	0	29	1	1	0	2	1	0	2	0
211	0	20	1	1	0	2	1	0	2	0
932	0	26	0	1	1	2	2	1	2	0
593	0	21	1	1	0	1	1	1	2	0
555	0	26	1	1	1	2	1	1	2	0
373	0	29	1	1	0	2	1	0	2	0
844	0	29	1	0	0	2	1	1	2	0
1188	0	19	1	0	1	1	1	0	2	0
544	0	8	1	1	0	2	2	1	2	0
490	0	29	1	1	0	1	1	1	2	0
905	0	25	1	1	0	1	2	1	3	0
937	0	29	0	1	1	2	2	0	2	0
1047	0	29	0	0	0	2	1	0	1	0
923	0	29	1	0	1	2	2	0	1	0

956	1	5	1	0	0	3	2	1	3	0
309	0	24	1	1	0	1	1	0	2	0
1180	0	29	1	1	0	1	2	1	2	0
1274	0	29	0	0	1	1	1	0	2	0
166	0	29	1	1	0	3	1	0	2	0
217	0	21	1	1	0	1	1	0	2	0
1345	0	29	1	1	0	2	1	0	1	0
581	1	2	1	0	1	1	3	0	2	0
72	0	29	1	1	0	1	1	1	1	0
588	0	28	1	0	0	1	1	0	2	0
141	0	29	1	1	0	2	1	1	2	0
722	1	2	1	0	1	1	2	1	3	1
859	0	25	0	0	0	1	1	1	2	0
153	0	29	1	1	0	1	2	0	2	0
294	0	25	1	1	0	2	1	0	2	0
277	1	2	0	1	0	1	2	1	3	0
41	0	25	1	1	1	2	2	1	2	0
431	1	8	0	0	1	1	1	1	3	0
90	0	19	1	1	0	1	1	0	1	0
316	0	29	0	1	0	1	1	0	2	1
1273	0	29	1	1	1	1	1	0	2	0
528	0	29	1	0	0	2	1	0	2	0
1161	1	6	0	0	0	1	2	1	3	0
774	0	26	1	1	0	1	1	0	2	0
747	0	12	1	1	0	2	1	1	2	0
456	0	26	1	1	0	2	2	0	2	0
598	0	22	1	1	0	2	1	1	2	0
1063	0	29	0	0	0	1	2	1	2	0
1204	0	29	1	0	0	1	1	0	2	0
752	0	29	1	1	0	2	1	0	2	0
1257	0	26	1	0	0	1	1	0	2	0
374	0	29	1	1	0	2	1	0	2	0
34	0	18	1	1	0	1	2	1	1	0
516	1	2	1	0	0	1	3	1	3	1
13	0	26	1	1	1	1	1	0	2	0
69	0	28	1	1	0	2	2	0	2	0
755	0	15	1	1	0	1	2	1	2	0
409	1	2	1	1	0	1	1	0	3	1

1364	0	29	0	1	0	1	2	1	3	0
1333	0	18	1	0	1	1	1	0	2	0
1133	0	29	1	1	0	2	2	1	2	0
928	0	22	1	1	0	1	1	1	2	1
1006	0	3	0	1	0	1	3	1	3	0
537	0	10	1	0	0	2	2	0	3	0
983	0	29	0	0	0	1	1	0	2	1
291	0	29	1	1	0	3	2	1	2	0
1341	0	29	1	0	1	2	2	0	3	0
671	1	4	1	1	1	2	1	1	2	0
121	0	6	0	1	0	1	1	0	1	0
1155	0	29	1	1	1	1	2	0	2	0
1203	0	23	1	0	1	1	2	1	2	0
1103	0	29	1	1	0	1	1	1	1	0
480	1	14	1	0	0	1	1	1	3	0
67	0	22	1	1	0	1	2	1	2	0
1014	0	21	1	0	0	2	1	1	2	0
1129	0	29	1	1	0	1	1	1	2	0
165	0	20	1	1	0	1	3	0	2	1
1075	0	29	0	0	1	2	1	1	2	0
1118	0	27	1	1	1	1	1	1	2	0
1223	0	26	1	1	1	1	2	1	2	0
236	0	29	1	0	0	2	1	0	2	0
610	0	29	1	0	0	2	1	0	2	0
330	0	10	1	1	0	1	2	0	1	0
726	0	22	1	1	0	2	2	1	2	0
127	0	27	1	1	0	2	2	0	3	0
212	0	29	1	0	0	1	1	0	2	0
686	0	29	1	0	0	1	2	1	1	0
814	1	1	1	0	1	1	1	0	3	1
931	0	29	1	0	0	1	1	0	2	0
878	0	29	1	1	0	2	1	1	2	0
243	0	29	1	1	0	2	3	1	2	0
847	0	29	0	0	1	1	1	0	1	0
1158	0	29	1	1	0	1	2	0	2	0
1388	0	16	0	0	1	1	1	0	2	0
619	0	29	1	1	0	2	1	0	2	0
477	0	26	1	1	0	2	2	1	2	1

151	0	14	0	1	0	1	1	0	2	0
666	0	6	1	1	0	3	1	1	1	0
767	0	29	1	1	1	2	2	1	1	0
160	0	29	1	1	1	1	2	1	2	0
155	0	5	0	0	0	3	1	0	2	0
426	0	29	1	1	0	2	1	0	1	0
1029	0	28	0	1	1	1	2	0	3	0
326	0	29	1	1	0	1	2	1	2	0
1335	0	19	0	0	0	1	2	0	2	0
789	0	29	1	0	1	2	1	1	2	0
1288	0	28	1	1	0	2	2	0	2	0
985	0	29	0	0	0	1	1	0	2	0
39	0	8	1	1	0	1	2	0	2	0
822	0	29	1	0	0	1	1	0	1	0
986	0	18	0	1	0	2	2	1	2	0
137	1	1	1	0	0	1	2	1	2	0
455	0	21	1	0	0	1	2	1	2	0
589	0	28	1	0	0	1	1	0	1	0
1127	0	29	1	1	0	1	1	1	2	0
1386	1	3	0	1	0	2	2	1	3	1
196	0	29	1	1	0	2	1	1	2	0
680	0	7	1	0	0	2	1	0	1	0
500	0	9	1	1	0	2	1	0	1	0
1342	0	29	1	0	0	1	1	0	2	0
344	0	29	1	0	0	1	2	0	2	0
459	0	29	1	1	1	1	1	1	2	1
944	0	27	0	0	1	1	1	0	3	0
1044	0	29	0	1	0	1	1	1	2	0
872	0	29	1	0	1	3	2	1	2	0
1471	0	10	1	1	1	3	1	0	2	1
1209	0	14	0	1	0	2	1	0	2	0
1076	1	28	0	0	1	3	1	1	2	1
876	0	28	0	1	0	2	1	1	2	0
534	0	17	1	1	0	1	1	0	2	0
177	0	29	1	1	0	2	1	1	1	0
554	0	29	1	1	0	2	3	1	3	0
827	0	29	1	0	0	2	2	1	2	0
84	0	29	1	0	0	2	1	1	1	0

633	0	29	0	1	0	1	1	0	1	0
597	0	29	1	1	0	1	1	0	1	0
706	0	29	1	1	0	3	3	0	2	0
1356	0	29	1	1	0	2	2	1	2	0
710	0	29	0	0	1	1	1	0	2	0
712	0	29	0	0	0	2	1	1	2	0
672	0	20	1	1	0	1	2	0	2	0
1302	0	29	1	1	0	2	1	0	2	0
1021	0	9	0	1	1	2	3	1	2	1
398	0	11	1	0	0	2	1	0	2	0
1013	0	29	0	1	1	1	2	0	3	0
1411	0	13	1	0	0	2	1	1	1	0
381	0	29	1	1	1	3	2	1	3	0
40	0	25	0	0	0	1	2	1	2	0
522	0	21	1	1	0	2	1	0	2	0
473	0	29	1	1	0	1	3	0	2	0
200	0	28	1	1	0	1	1	1	1	1
1170	0	29	1	0	0	1	1	0	2	0
265	0	24	1	1	0	2	3	1	2	0
775	0	16	1	1	0	2	1	1	2	0
186	0	17	1	1	0	1	2	1	2	1
458	0	27	1	1	0	1	1	0	2	0
1197	0	29	1	1	0	1	1	1	1	0
831	0	29	1	1	1	1	3	1	2	1
1078	0	20	0	1	0	2	1	1	2	0
919	0	17	1	0	0	2	1	1	2	0
235	0	29	1	0	0	1	1	0	1	0
185	0	14	1	1	0	1	2	1	2	0
413	0	29	1	1	0	1	1	1	2	0
627	0	18	0	1	0	2	1	1	2	0
1298	0	9	1	0	0	1	1	0	2	0
1286	0	29	0	0	0	2	1	0	2	0
205	0	29	1	1	0	1	3	1	3	1
779	1	6	1	0	1	3	1	1	3	1
564	0	29	1	1	0	1	1	0	2	0
794	0	7	0	0	0	1	2	1	2	0
1395	0	29	1	0	0	1	1	0	2	0
1351	0	3	1	1	0	1	1	1	2	0

468	0	23	1	0	0	1	1	0	1	0
920	1	3	0	0	0	1	2	1	3	0
1083	1	22	0	1	0	2	2	0	2	1
457	0	1	0	0	0	1	1	0	2	0
617	0	20	0	1	0	2	1	1	1	0
357	0	29	1	0	0	1	1	0	2	0
279	0	29	1	1	0	1	2	1	2	1
270	0	29	0	1	0	1	1	0	1	0
1361	0	11	0	1	0	2	3	0	2	0
646	0	24	1	1	0	2	1	0	2	0
347	0	15	1	0	0	2	2	0	3	0
1174	0	13	1	1	1	2	1	1	2	0
218	1	1	0	0	0	2	2	0	2	1
618	0	29	1	0	0	1	2	1	2	0
698	0	29	1	1	0	1	2	1	2	0
337	0	25	1	1	1	2	1	0	2	0
797	0	16	1	1	0	2	1	0	2	0
1449	0	29	1	1	0	1	1	1	3	0
539	1	3	0	0	0	3	2	1	3	1
981	0	13	0	0	1	2	1	0	2	0
1463	0	29	1	0	0	1	1	0	2	0
724	1	15	1	1	0	1	2	1	2	1
1385	0	17	1	0	0	2	3	1	2	0
861	0	29	0	1	0	1	2	1	2	0
657	0	3	1	1	0	1	1	0	2	0
960	0	29	1	1	0	2	1	1	2	0
1270	1	2	1	1	0	2	1	0	2	1
660	0	20	0	1	0	1	2	0	2	0
163	0	21	1	1	0	1	2	1	2	0
238	0	20	1	1	0	1	1	0	1	0
578	1	14	1	0	0	1	2	0	2	0
1368	0	25	1	1	0	2	1	0	2	0
117	0	29	1	1	0	1	1	0	1	0
648	0	8	1	1	0	2	1	0	2	0
1079	0	23	0	1	1	2	2	0	2	0
1312	0	29	1	0	0	1	2	1	2	0
557	0	28	1	0	0	2	2	1	2	0
1476	0	7	0	0	0	1	1	1	1	0

1179	0	16	1	0	0	2	1	0	1	0
957	0	27	0	0	0	2	1	0	2	0
873	0	29	0	1	1	3	2	1	2	0
688	0	14	1	0	1	1	2	1	2	0
757	0	19	0	1	0	2	3	1	3	1
988	0	10	0	0	0	1	1	0	2	0
447	0	29	1	0	0	2	1	0	2	0
1148	0	29	1	1	0	1	1	0	2	0
821	0	11	1	1	0	2	1	0	2	0
1287	0	25	1	1	1	1	2	1	2	0
711	0	26	0	0	0	2	1	0	1	0
349	0	29	1	1	0	1	3	1	2	0
1319	0	29	1	1	0	1	1	0	2	0
386	0	29	1	1	1	1	1	0	2	0
1186	0	25	0	0	1	2	1	0	1	0
24	0	29	1	0	0	1	1	1	1	0
945	0	26	0	1	0	2	1	1	2	0
1023	0	27	0	0	1	2	2	1	1	0
1175	0	24	1	0	0	1	2	1	2	0
1375	1	6	1	1	0	2	3	1	3	1
170	0	2	1	0	0	1	1	0	2	0
1309	0	25	1	1	1	1	2	1	2	0
422	0	4	0	0	0	1	1	0	2	0
508	0	29	1	1	0	1	2	1	2	0
64	0	13	1	1	0	2	2	0	1	0
80	0	29	1	0	0	2	1	0	2	0
548	0	29	1	1	0	2	2	0	2	0
987	0	23	0	1	1	1	3	1	3	0
475	1	2	1	0	0	3	2	1	2	1
1387	0	22	1	1	0	1	2	0	2	0
765	0	19	1	0	0	3	1	0	2	0
479	1	5	1	0	0	1	2	0	2	0
1156	0	23	1	0	0	1	2	0	2	0
791	0	29	0	0	1	1	1	0	2	0
1437	0	11	1	1	0	2	1	1	2	0
807	0	29	1	1	0	1	1	1	2	0
292	0	5	1	1	0	1	1	0	2	0
297	0	29	1	1	0	2	2	0	2	0

860	0	27	1	1	1	2	2	1	2	0
605	0	29	1	1	0	2	2	0	2	0
637	0	22	1	0	0	2	1	0	2	0
1405	0	29	1	1	1	3	1	1	3	1
1210	0	10	1	0	0	1	1	0	2	0
1310	0	27	1	0	0	2	2	0	2	0
1057	0	29	0	0	1	2	1	1	2	0
83	0	29	1	1	1	1	1	1	2	0
866	0	13	1	1	0	2	1	1	2	0
1417	0	3	1	1	0	1	1	0	1	0
1212	0	23	1	0	0	2	1	0	2	0
76	0	29	1	1	0	1	3	1	3	0
1138	1	11	1	0	1	2	1	1	1	0
1054	0	12	0	1	0	2	2	0	2	0
946	0	14	1	1	0	2	2	1	2	0
1401	0	27	0	0	0	1	1	0	1	0
323	0	25	0	1	0	2	3	1	2	0
115	0	29	1	1	1	2	2	1	2	0
850	0	29	1	1	0	2	1	1	2	0
608	0	29	1	1	1	1	2	1	2	0
682	0	29	1	1	1	2	1	1	2	0
1470	0	25	1	0	1	2	1	0	2	0
1140	0	27	1	0	0	2	1	0	2	0
397	0	29	1	1	0	2	2	0	2	0
989	0	20	0	1	1	2	2	0	2	0
392	1	2	1	0	0	1	2	0	3	1
1445	0	29	1	0	0	1	3	1	2	1
744	0	21	1	1	0	1	1	0	3	0
1360	0	22	1	0	0	1	1	0	2	0
106	0	20	1	0	0	2	1	0	2	0
11	0	29	1	1	0	3	2	1	2	0
625	1	1	0	0	1	3	3	0	3	0
1216	0	23	1	1	0	2	1	1	2	0
403	1	1	1	0	0	2	2	1	2	1
461	0	29	1	1	0	2	2	1	2	0
1423	0	29	1	0	0	1	2	1	2	0
31	0	24	1	1	0	1	1	0	1	0
94	1	2	0	0	1	1	3	1	2	1

16	0	23	1	1	0	2	2	1	2	0
178	0	23	1	1	0	2	3	1	3	0
1217	0	29	1	0	0	2	1	0	2	0
524	0	26	1	1	0	1	1	0	2	0
924	0	29	0	0	0	1	1	0	2	0
204	0	20	1	1	0	1	2	0	2	0
1443	0	5	1	0	0	1	1	0	2	0
1261	0	1	0	0	0	2	2	1	2	0
384	0	25	1	1	0	1	1	1	2	0
315	1	17	1	1	1	1	1	1	2	1
259	0	3	0	1	0	2	1	0	2	0
494	0	29	1	1	1	1	2	0	1	0
1072	0	29	0	1	0	1	1	0	2	0
1016	0	20	0	0	0	1	1	0	2	0
10	0	12	1	0	0	1	1	0	1	0
1323	0	23	1	1	0	1	2	1	2	0
402	0	29	1	0	0	1	2	0	2	0
108	0	16	1	1	0	1	1	1	2	0
8	0	29	0	0	0	2	1	0	1	0
626	1	11	1	0	1	1	3	1	2	1
261	0	10	1	1	0	1	1	0	2	0
541	0	29	0	0	0	1	1	1	2	0
1346	0	29	1	1	1	2	1	1	1	0
1373	0	29	1	0	1	1	1	0	2	0
282	1	3	0	1	1	1	2	1	3	1
1147	0	29	0	0	0	2	1	0	2	0
696	1	4	1	1	0	1	2	1	2	1
667	0	22	1	0	0	1	2	0	3	0
990	0	24	0	0	0	1	1	1	2	0
452	0	29	1	1	0	1	2	0	2	0
856	0	29	1	1	0	3	2	1	2	0
622	0	26	1	0	0	2	2	1	2	0
1145	0	28	1	1	0	1	1	1	2	0
1060	0	29	0	1	0	3	1	0	1	0
1235	0	29	1	1	1	1	3	1	3	0
891	1	2	0	0	0	3	2	1	3	1
1034	0	28	0	1	0	1	2	1	2	0
1461	0	28	1	0	0	1	1	0	1	0

793	0	29	1	1	0	1	2	0	2	0
1475	1	2	1	1	0	2	2	1	2	1
278	0	29	1	1	0	2	2	1	2	0
241	0	29	1	1	0	2	2	1	2	0
1214	0	18	1	1	0	2	1	1	2	0
679	0	6	0	0	0	2	1	1	2	0
37	0	29	1	1	0	2	2	0	2	0
1394	0	29	1	1	0	1	2	0	2	0
566	0	10	1	1	0	2	1	0	2	0
19	0	29	1	0	0	1	2	0	2	0
378	0	29	1	1	0	2	1	0	2	0
549	0	29	1	1	1	2	3	1	2	0
48	0	28	1	1	0	2	1	0	1	0
464	0	29	1	1	0	2	2	1	2	0
393	0	12	1	1	0	1	1	0	2	0
670	0	15	1	1	0	1	2	1	2	0
311	1	9	1	1	0	2	2	0	2	0
189	0	29	1	0	0	2	1	0	1	0
38	0	7	1	1	0	3	2	0	2	0
319	0	29	0	0	0	2	1	0	1	0
1102	0	17	1	1	0	1	1	1	2	0
846	0	29	0	0	1	2	1	0	1	0
120	1	10	1	1	1	1	1	1	2	1
1308	0	3	1	1	0	1	3	1	2	0
441	0	23	1	1	0	1	1	0	2	0
599	0	29	1	1	0	2	1	0	2	0
1425	0	29	1	0	0	2	2	1	2	0
714	0	29	1	1	0	1	1	1	2	0
677	0	29	0	1	1	2	2	1	2	0
81	0	17	1	1	0	1	1	0	3	1
134	0	29	1	1	0	2	2	1	2	0
424	0	29	1	1	0	1	2	0	2	0
756	0	24	1	1	0	2	1	1	2	0
6	0	20	0	1	0	2	2	0	2	0
879	0	29	1	1	0	2	2	1	2	0
668	0	15	1	1	0	1	2	0	3	0
49	0	29	1	0	1	3	2	0	2	0
193	0	20	1	0	0	1	1	0	3	0

1459	0	21	1	0	0	3	1	1	2	0
1328	0	14	0	0	0	1	1	0	2	0
303	1	4	0	0	0	2	3	0	2	1
898	1	3	1	0	0	2	2	1	3	1
190	1	9	0	1	1	3	1	1	2	1
191	0	24	1	0	1	1	2	0	2	0
446	0	29	1	1	0	1	2	0	2	0
119	0	26	1	1	0	1	1	0	2	0
817	0	29	1	1	0	1	1	0	2	0
61	0	29	1	0	1	1	1	0	1	0
1259	1	21	0	0	0	1	1	1	2	0
930	0	29	0	0	0	2	1	0	2	0
950	0	29	0	0	0	1	2	1	2	0
1256	0	10	0	0	0	2	1	0	2	0
1357	0	29	1	1	0	1	1	0	1	0
758	0	29	1	0	0	1	1	1	2	0
993	0	29	1	0	0	1	1	0	2	0
947	0	7	0	1	0	1	1	0	2	0
690	0	29	1	1	0	2	3	1	2	0
251	0	29	1	1	0	1	2	1	2	1
560	0	29	1	1	0	2	2	0	2	0
643	0	29	1	1	1	1	2	1	2	0
545	1	1	0	0	1	3	3	1	3	1
1124	0	29	1	1	0	2	2	1	3	0
162	1	19	1	0	0	2	2	1	2	0
576	0	29	1	0	0	1	1	1	1	0
168	0	29	1	1	0	1	2	1	2	0
788	0	23	1	0	0	1	3	0	2	0
78	0	21	1	1	0	1	3	1	2	0
1115	0	29	1	1	1	2	2	1	2	0
445	0	29	1	0	0	1	1	0	2	0
995	0	18	1	0	0	1	2	0	3	0
95	0	24	1	1	0	2	1	0	2	0
918	0	7	0	0	0	1	3	1	3	1
1243	0	29	0	0	0	2	2	1	2	0
379	0	29	1	1	0	2	2	1	2	0
342	0	21	1	1	0	2	3	1	2	0
221	0	29	0	0	0	1	1	0	2	0

1126	0	15	1	0	0	1	1	0	2	0
161	0	29	1	1	0	2	2	0	3	0
620	1	20	1	1	0	1	2	1	2	0
448	0	29	1	1	0	2	3	1	2	0
242	0	29	1	0	0	2	2	0	2	0
693	1	13	1	1	0	1	2	0	2	1
927	0	20	0	0	0	2	1	0	2	0
1363	0	29	1	1	0	1	3	1	2	0
968	0	19	1	1	1	2	1	1	1	0
536	0	17	1	1	0	2	2	0	1	0
828	0	26	1	0	1	2	2	0	2	0
926	0	29	1	1	1	2	1	0	1	1
407	0	29	0	0	1	1	1	0	2	0
229	0	29	1	1	0	1	1	0	2	0
224	0	29	1	0	0	2	1	1	2	0
785	0	23	0	0	0	2	1	0	1	0
474	0	29	1	1	0	1	3	0	2	0
699	0	15	1	0	0	2	1	0	2	0
1070	0	25	0	1	0	1	1	0	2	0
171	0	29	1	1	0	1	1	0	1	0
23	0	4	1	1	0	1	3	1	2	0
1258	0	29	0	0	0	1	1	0	1	0
484	0	29	1	0	0	1	1	1	2	0
301	0	1	1	0	0	3	1	1	3	0
1236	0	29	1	1	0	2	2	0	2	0
79	0	12	1	0	0	1	1	0	2	0
511	0	5	1	1	0	2	2	0	2	0
507	0	28	1	1	0	1	2	1	2	0
164	0	29	1	1	0	1	3	1	2	0
237	0	29	1	1	0	2	1	1	2	0
579	0	27	1	1	0	2	1	0	2	0
1194	0	27	1	1	0	2	1	1	2	0
929	0	29	1	1	0	2	3	1	2	0
1207	0	28	0	1	0	2	1	1	2	0
493	1	4	1	1	0	3	1	1	2	0
730	0	29	1	1	0	2	1	1	1	0
796	0	29	1	0	0	1	2	0	1	0
209	0	29	1	1	0	1	1	0	2	0

1069	0	29	0	1	0	1	2	1	3	0
1306	0	29	0	0	0	2	1	0	2	0
358	0	29	0	0	0	1	3	1	2	0
650	0	29	1	1	0	1	1	1	2	0
877	0	29	0	1	0	1	1	1	2	0
978	0	24	1	1	0	1	1	0	2	0
1416	0	28	0	0	0	1	1	0	2	0
904	0	22	0	1	0	1	1	0	2	0
129	0	24	1	1	0	2	1	1	2	0
848	0	29	1	1	0	2	1	0	2	0
886	0	29	1	1	1	1	1	1	3	0
450	0	27	1	1	0	1	2	0	2	0
232	0	10	1	0	0	1	1	0	2	0
334	0	29	1	1	0	2	1	0	2	0
396	0	21	1	0	0	1	2	0	2	0
1042	0	20	0	1	0	1	2	0	2	0
1450	0	29	1	1	0	2	2	0	2	0
639	0	29	0	0	0	1	1	0	2	0
974	0	29	1	1	1	3	2	1	3	0
264	0	29	1	1	0	2	2	1	1	0
697	0	29	1	0	1	3	1	0	1	0
201	0	17	1	0	0	1	2	0	2	0
52	0	20	1	1	1	1	1	0	2	0
225	1	6	1	1	1	3	2	1	3	1
1378	0	17	1	1	0	1	2	0	2	0
1332	0	15	1	0	0	1	1	0	2	0
770	0	29	1	0	1	1	1	1	2	0
577	0	19	1	1	0	1	1	0	1	0
1268	0	25	1	1	1	1	2	0	2	0
903	0	29	0	1	1	1	3	1	3	0
1131	0	21	1	0	0	1	1	0	1	0
20	0	29	0	1	0	2	1	1	2	0
206	0	29	1	0	0	3	1	0	1	0
124	0	27	1	1	0	1	1	0	1	0
592	0	29	1	0	0	1	2	0	2	0
1291	0	23	1	1	0	1	1	0	1	0
740	0	26	1	1	0	1	1	0	2	0
45	0	21	0	0	1	2	3	0	3	0

332	0	29	1	1	0	2	2	0	2	0
281	0	18	1	1	0	2	2	1	2	0
91	0	19	1	1	0	1	1	0	2	0
653	0	29	1	0	1	1	1	0	2	0
138	0	21	1	1	0	1	1	0	2	0
606	0	29	1	1	0	1	3	0	3	0
1366	0	21	1	1	0	2	1	0	2	0
425	0	22	1	1	0	2	2	0	2	0
780	1	27	1	0	0	2	3	1	2	0
1134	0	29	1	1	1	1	2	1	2	0
839	0	27	1	1	0	1	1	1	2	0
271	0	29	1	1	0	1	1	0	2	0
595	0	29	1	1	0	1	1	0	1	0
1408	0	18	1	0	0	1	1	1	1	0
167	0	29	1	1	0	2	1	0	1	0
499	0	29	1	1	0	2	2	0	2	0
255	1	11	1	1	0	1	3	1	2	0
980	1	1	0	0	1	3	2	0	3	1
634	0	29	0	1	0	2	2	1	2	0
902	0	29	0	1	0	2	2	1	3	0
71	0	29	1	1	0	1	1	0	1	0
772	0	20	1	1	0	1	2	1	3	0
1065	0	29	0	0	0	1	2	0	2	0
776	0	14	1	0	0	2	1	1	2	0
437	0	16	1	1	0	2	1	0	2	0
1224	0	29	1	1	0	2	1	1	2	0
1321	0	20	1	1	1	1	3	0	2	0
896	1	3	1	0	1	2	3	1	3	1
1181	1	2	0	1	1	1	3	1	2	1
750	0	29	1	0	0	1	1	1	2	0
1264	0	18	1	1	0	2	1	1	1	0
412	0	29	1	1	0	1	1	1	2	0
1120	0	19	1	0	1	1	2	1	2	0
1338	0	9	1	0	0	1	1	1	2	0
673	0	29	1	1	0	2	1	1	2	0
628	0	29	1	1	0	1	2	1	2	0
46	0	29	1	0	0	1	1	1	2	0
1205	0	29	1	1	0	2	3	1	3	1

531	0	29	1	0	0	2	1	1	2	0
1005	0	27	0	0	0	1	1	0	1	0
1369	0	29	1	1	0	1	1	1	2	0
972	0	29	0	1	1	2	1	0	2	0
220	1	4	1	1	1	2	3	1	2	0
692	0	29	1	1	1	2	1	0	2	0
222	1	2	1	1	0	2	3	1	2	0
836	0	29	1	0	0	2	1	0	2	0
991	0	29	0	1	0	1	1	1	2	0
1460	0	29	1	0	0	2	1	1	1	0
122	0	8	1	1	0	1	1	0	2	0
331	0	18	1	0	0	2	2	1	1	0
502	0	7	1	1	0	1	1	0	1	0
996	0	15	0	0	0	1	1	1	2	0
787	0	16	1	0	1	1	1	0	2	0
1200	0	21	1	0	0	2	1	0	2	0
743	0	26	1	1	0	1	1	0	2	0
1056	0	23	0	1	0	2	1	0	2	0
829	0	26	1	0	0	2	1	0	2	0
169	0	29	1	1	0	3	2	1	2	1
729	0	29	1	0	0	1	1	0	2	0
1372	0	19	1	0	0	2	1	0	2	0
561	1	1	1	0	1	1	2	0	2	0
341	0	29	1	1	1	1	1	1	2	0
1397	0	10	1	0	0	1	2	1	3	0
320	0	29	0	0	0	1	1	1	2	1
504	1	10	1	1	0	1	1	0	3	1
1431	1	25	0	0	1	2	2	0	3	0
2	1	8	0	0	1	3	3	1	2	0
786	0	2	0	0	0	1	1	0	2	0
921	0	29	0	0	1	1	2	1	2	1
1162	0	29	1	1	0	1	2	1	2	0
111	0	27	1	1	0	1	1	1	2	0
855	0	12	0	0	0	1	2	1	2	1
1176	0	13	0	0	0	2	1	0	2	0
1068	1	20	1	1	1	2	1	1	3	0
449	1	3	1	0	0	1	3	1	3	1
951	0	29	0	0	1	1	2	1	2	0

523	0	6	1	0	0	2	2	0	2	0
719	0	29	1	0	0	1	1	0	2	0
335	0	29	1	0	0	1	1	0	2	0
56	0	29	1	1	0	2	1	0	2	0
1402	0	27	0	0	0	2	2	1	2	0
1362	1	12	1	1	0	2	2	0	1	1
207	1	10	0	0	1	2	2	1	2	1
436	1	5	0	0	1	3	1	1	2	1
601	0	29	1	0	0	1	1	1	2	0
1193	0	21	1	1	0	1	1	0	2	0
387	0	29	1	1	0	2	1	1	2	0
263	0	29	1	1	0	1	1	1	2	0
68	0	29	1	1	0	1	2	1	2	0
1169	1	9	1	1	0	1	1	1	2	0
1164	0	29	1	1	0	3	2	1	2	1
565	0	14	1	0	0	2	2	0	2	0
1266	0	29	0	1	0	2	1	0	1	0
792	0	29	0	1	0	2	1	1	2	0
742	0	8	1	1	0	2	2	0	3	0
897	0	14	0	1	0	1	1	0	1	0
835	0	29	1	0	0	3	1	0	1	0
1183	0	20	0	0	0	1	2	0	3	0
586	0	29	1	1	0	1	1	0	1	0
256	0	29	1	1	0	1	1	1	2	0
1218	0	13	1	1	0	2	1	0	2	0
471	0	29	1	1	0	1	1	0	1	0
88	0	16	0	0	0	1	1	0	2	0
416	0	2	1	1	0	1	1	0	2	0
1410	0	29	1	1	0	2	2	0	1	0
1442	0	29	1	0	0	1	1	0	2	0
1113	0	29	0	1	0	1	2	1	3	0
1396	0	27	1	0	0	2	1	0	2	0
700	0	13	1	0	0	1	1	0	2	1
287	0	3	1	1	0	1	2	0	2	0
685	0	29	1	1	0	2	2	1	3	0
1315	0	19	1	1	1	2	3	1	3	0
280	1	18	1	1	0	2	2	1	2	0
512	0	29	1	1	0	3	3	1	1	0

803	0	29	1	0	1	1	1	1	1	0
1377	0	29	0	0	0	3	1	0	1	1
778	0	29	1	0	0	2	2	1	2	0
1195	0	28	1	0	1	2	1	0	1	0
328	0	12	1	0	0	2	1	0	2	0
172	0	24	1	1	0	1	2	0	3	0
298	0	21	1	0	0	1	1	0	1	0
1412	0	29	1	0	1	1	1	0	1	0
1104	1	6	0	1	0	3	2	1	2	1
678	0	7	0	0	0	1	1	1	2	0
529	0	29	1	1	0	2	3	1	2	0
1272	0	24	0	0	1	1	1	0	1	0
1144	0	29	1	1	0	2	2	1	1	0
741	0	23	1	1	1	2	2	1	2	0
365	0	29	1	0	0	1	2	1	2	0
591	0	29	1	1	0	1	1	0	2	0
841	1	1	1	0	1	3	2	0	3	0
227	0	29	1	1	1	1	3	1	2	0
1121	0	29	0	0	0	2	2	0	3	0
1143	0	20	1	1	0	1	2	1	2	0
1469	0	29	1	1	0	2	2	1	2	1
1292	0	29	1	1	0	2	2	0	2	0
535	0	29	1	1	0	2	2	0	2	0
1114	0	15	0	0	0	3	1	1	2	0
112	0	29	1	1	0	3	1	1	2	0
1407	0	29	1	1	0	2	2	0	3	0
93	0	26	0	1	0	2	2	0	2	0
489	0	9	0	0	0	2	2	1	3	1
1344	0	29	1	1	0	2	1	1	3	0
631	1	2	0	0	1	1	2	0	2	0
1088	1	3	0	1	0	1	3	0	1	0
482	0	28	1	0	0	1	1	1	2	0
248	0	18	1	1	0	1	1	0	1	0
105	0	23	1	0	0	3	3	1	2	0
999	0	29	0	1	0	2	1	0	2	0
1018	0	29	1	1	0	1	1	0	2	0
1055	0	17	0	1	0	1	1	0	2	0
1399	0	29	1	1	1	1	3	1	2	0

312	0	22	1	1	0	2	2	1	2	0
562	0	26	0	1	0	2	2	0	2	0
139	0	29	1	1	1	1	3	1	3	1
1041	0	14	0	0	0	1	2	1	2	0
481	1	1	1	0	1	3	3	0	3	1
250	0	29	1	1	0	1	2	1	2	1
971	0	29	1	1	1	1	1	0	2	0
1234	0	29	1	0	0	1	1	0	2	0
1238	0	16	0	1	0	1	2	1	2	1
1313	0	29	1	0	0	1	1	0	2	0
1392	1	7	1	1	0	3	2	1	2	0
1324	0	29	0	0	0	1	3	0	1	0
99	0	29	1	1	1	1	1	1	2	1
98	0	27	1	1	0	1	1	0	2	0
58	0	25	0	1	1	1	2	0	3	0
1441	0	29	1	1	0	3	1	0	2	0
525	0	27	1	0	0	2	1	0	1	0
933	1	5	1	1	0	1	2	1	3	0
258	1	8	1	0	0	1	1	0	2	0
635	1	16	1	1	0	2	2	1	2	0
262	0	27	1	1	0	1	2	1	2	0
925	0	29	0	0	0	2	1	1	2	0
818	0	20	1	0	0	2	1	0	2	0
948	0	29	0	0	0	2	1	1	2	0
199	1	5	1	0	1	1	2	1	3	0
1336	0	29	1	1	0	2	1	0	1	0
849	1	10	0	1	1	3	1	1	2	0
234	0	24	1	1	0	2	1	0	1	0
768	0	7	1	1	0	2	2	0	2	0
55	0	29	1	1	0	1	1	0	2	0
973	1	2	0	0	0	1	3	1	3	0
753	0	23	1	0	0	1	1	0	1	0
488	1	2	0	0	1	1	2	0	2	1
1246	0	21	1	0	1	1	1	0	2	0
1303	0	29	1	0	0	2	1	0	2	0
997	0	18	0	1	0	1	1	0	1	0
1350	0	17	0	1	1	2	3	1	2	0
1251	1	22	0	1	0	2	3	1	2	0

1116	0	29	0	0	0	1	2	1	3	0
1242	0	5	0	0	0	1	1	1	3	0
1260	0	29	1	0	0	1	1	0	2	0
74	0	23	1	0	0	2	2	0	2	0
851	0	29	0	0	1	1	1	1	1	0
329	0	29	1	0	0	1	2	1	3	0
1248	1	10	0	0	1	1	2	1	3	0
339	0	29	1	1	0	1	1	0	2	0
761	0	29	1	1	0	3	2	1	3	0
247	0	18	1	1	0	1	1	0	1	0
370	0	29	1	1	0	1	1	0	2	0
530	1	14	1	0	0	1	1	0	2	0
44	1	5	0	0	0	2	2	1	2	0
533	0	29	1	1	0	1	3	1	2	0
1326	0	26	1	1	0	2	2	1	2	0
376	0	29	1	1	0	3	1	1	2	0
1314	0	29	1	1	0	1	1	0	2	0
806	0	29	1	1	0	2	1	0	2	0
1317	0	29	1	0	0	1	1	0	2	0
887	0	28	1	1	0	1	1	1	2	0
194	0	19	1	1	0	2	3	1	2	0
594	0	29	1	1	0	2	2	1	2	0
460	0	20	1	1	0	2	1	0	2	0
1031	1	1	0	0	0	2	3	0	3	0
77	1	3	0	0	1	1	2	1	3	1
1215	0	22	0	0	0	1	2	0	2	0
691	0	29	1	0	0	2	1	0	2	0
658	1	24	0	0	0	1	1	0	2	0
266	0	14	1	1	0	1	1	0	2	0
713	0	4	1	1	1	2	1	1	2	0
355	0	10	1	1	0	2	2	0	1	0
470	0	29	1	1	0	2	1	0	2	0
745	0	5	1	0	0	1	1	1	3	0
1465	0	29	1	1	1	1	2	0	2	0
1167	0	2	1	1	0	2	2	1	2	0
874	0	25	0	1	1	2	2	1	3	0
380	0	29	1	1	0	2	2	1	2	0
804	0	29	1	1	0	2	2	1	3	0

5	0	9	1	1	1	1	3	1	2	1
469	0	29	1	1	0	1	3	1	2	0
1198	0	29	1	1	0	2	1	1	2	0
612	0	24	1	1	0	1	1	0	1	0
411	0	27	1	1	1	1	1	0	2	0
1390	0	12	1	0	0	1	1	0	2	0
655	0	14	0	0	0	1	1	0	1	0
615	0	29	1	1	0	2	2	1	2	0
1285	0	17	1	1	0	2	1	0	2	0
391	0	29	1	0	0	2	2	0	1	0
290	0	29	1	1	0	1	1	1	2	0
833	0	29	1	1	0	1	2	1	3	0
466	0	14	1	1	0	2	3	1	3	0
815	0	29	1	0	0	1	1	0	2	0
890	0	29	0	1	0	2	1	0	2	0
1202	0	26	1	0	0	2	1	0	2	0
813	0	20	1	1	1	2	3	1	2	0
647	0	29	1	1	0	2	1	1	2	0
478	0	29	1	1	0	1	2	0	1	0
321	0	22	1	1	0	1	2	1	2	0
9	0	29	0	0	0	2	1	0	2	0
941	0	8	0	0	0	2	1	0	2	0
642	0	29	1	0	0	1	1	0	2	0
1299	0	29	1	1	0	3	2	1	1	0
148	0	4	1	1	0	2	2	0	2	0
651	1	4	0	1	0	1	1	0	2	0
18	0	25	1	0	1	1	3	1	3	0
1062	0	22	0	1	0	1	3	1	2	0
1454	0	29	1	1	0	2	1	0	2	0
1250	0	29	0	0	0	3	1	0	2	0
1190	0	25	1	1	0	2	2	0	2	0
383	0	29	1	1	0	1	2	0	2	0
551	1	1	0	0	1	1	2	0	2	1
1354	0	29	1	1	0	1	1	1	2	0
1043	0	4	0	1	1	1	2	1	2	0
812	0	29	1	0	0	2	1	0	2	0
1329	0	29	1	0	0	1	2	1	2	0
1030	0	19	0	0	1	2	2	0	2	0

1453	0	20	1	0	0	1	2	1	2	0
421	0	29	1	1	0	2	2	1	2	0
444	0	29	1	0	0	2	2	0	3	0
29	0	28	1	1	0	1	1	0	1	0
781	0	29	0	0	0	1	1	0	1	0
1109	0	15	1	0	0	2	2	0	2	0
1027	1	23	0	1	1	1	1	1	3	0
42	0	21	1	1	0	1	1	0	2	0
1327	0	29	0	1	0	1	1	0	2	0
1004	0	29	0	0	1	2	1	1	2	0
773	0	29	1	1	1	1	3	1	2	0
1171	0	29	0	0	0	2	1	0	2	0
352	1	8	1	0	1	3	3	1	2	0
1428	1	2	0	0	0	2	3	1	3	0
451	0	28	1	0	0	1	1	0	2	0
863	0	29	0	0	0	1	1	0	1	0
1112	0	29	1	0	0	1	1	0	2	0
401	1	20	0	1	1	3	1	0	2	0
1048	0	25	0	1	1	1	1	1	2	0
611	0	28	0	1	0	1	2	0	2	0
654	0	12	1	0	0	3	1	0	3	0
867	1	1	0	0	1	1	2	1	3	1
375	0	29	1	1	0	3	1	1	2	0
884	0	29	0	1	1	2	3	1	3	0
1220	0	29	1	1	0	2	2	0	1	0
830	0	29	0	1	1	2	2	0	2	0
520	0	17	1	1	0	1	1	0	2	0
749	0	29	1	0	0	1	1	1	2	0
86	0	29	1	1	0	3	1	1	1	0
434	0	19	1	1	0	2	2	1	2	0
17	0	10	0	1	0	2	2	1	2	0
1241	0	29	0	0	1	1	1	0	2	0
485	0	25	1	1	0	1	1	0	1	0
723	0	29	1	0	0	1	1	1	1	0
883	0	27	1	0	1	1	1	1	2	0
1283	0	29	0	0	1	2	2	0	2	0
1002	0	29	1	1	0	3	1	1	3	0
1422	0	29	0	0	0	1	1	0	2	0

35	0	24	1	1	1	1	2	1	2	0
538	0	29	1	1	0	1	2	0	2	0
36	0	29	0	1	0	2	1	0	2	0
503	0	29	1	0	0	2	2	0	2	0
1430	1	1	0	0	1	2	2	0	3	0
28	1	9	1	0	1	2	2	1	2	0
310	0	9	0	1	0	2	2	0	2	0
1420	0	27	0	0	1	1	1	0	2	0
285	0	29	1	1	0	1	1	1	2	0
1464	0	23	1	1	1	1	2	1	2	0
802	0	14	1	1	1	1	2	1	2	1
269	0	29	1	1	1	1	1	1	2	0
372	0	29	1	1	0	2	3	1	2	0
517	0	29	1	1	0	1	1	0	2	0
661	0	29	1	1	1	1	1	0	2	0
728	0	29	1	1	1	3	1	1	3	0
820	0	21	1	0	0	2	1	0	2	0
1074	0	26	0	1	0	1	3	1	2	0
584	0	29	1	1	0	1	1	0	2	0
126	0	29	1	1	1	1	1	0	2	0
1330	0	8	0	0	0	1	1	0	2	0
152	1	5	1	1	0	2	3	1	1	0
75	0	19	1	0	0	1	1	0	1	0
769	0	29	1	0	0	1	2	1	2	0
1436	0	27	1	1	0	2	1	0	2	0
1379	0	12	0	0	0	2	3	1	1	1
304	0	29	1	0	0	3	1	0	2	0
994	0	8	0	1	0	2	1	1	2	1
669	0	10	1	0	1	1	1	0	3	0
100	0	12	1	1	0	1	1	1	2	0
25	0	5	1	0	0	1	2	0	1	0
614	0	29	1	0	1	1	1	0	2	0
180	0	3	0	0	0	2	1	0	2	0
293	0	29	1	1	0	1	3	1	2	0
1252	0	29	0	1	0	1	2	0	2	0
1447	0	28	1	0	0	2	2	0	2	0
547	0	29	0	1	0	1	3	0	2	0
343	0	23	1	0	1	2	3	0	3	0

976	1	2	0	0	1	3	2	1	3	1
1191	0	15	1	0	0	2	1	0	2	0
433	1	17	1	1	1	1	2	1	2	0
889	0	21	1	1	1	1	1	0	1	0
366	0	18	1	1	1	2	1	0	2	0
1400	0	29	1	0	0	1	1	0	2	0
1300	0	29	0	0	1	1	2	0	2	0
959	0	18	1	0	0	1	1	0	2	0
1249	1	6	1	0	1	3	1	1	3	1
935	0	18	1	1	1	1	2	0	3	0
1334	1	20	0	0	1	1	1	1	2	1
652	0	29	1	1	0	2	2	1	1	1
1263	1	17	0	0	1	1	1	1	2	0
215	0	29	1	1	1	2	2	1	2	0
103	0	29	1	1	0	2	1	0	2	0
400	1	1	0	1	1	3	1	0	3	1
916	0	18	0	0	0	3	1	1	2	0
1028	0	27	1	0	1	1	1	0	2	0
158	0	29	0	0	0	1	1	0	1	0
156	0	29	1	1	0	2	2	0	2	0
587	0	23	1	1	0	1	1	0	1	0
286	0	22	1	0	0	2	1	1	2	0
423	0	27	1	1	0	2	1	0	2	0
1130	0	29	1	0	0	2	1	0	2	0
430	0	12	1	1	0	1	2	0	3	0
1046	0	29	0	0	0	1	1	0	2	0
1036	0	27	0	1	0	1	1	0	1	0
540	0	26	0	0	1	1	1	0	2	0
385	0	22	1	1	0	3	1	0	2	0
1059	0	29	0	1	0	2	2	1	2	0
1406	0	20	1	1	0	1	1	0	2	0
676	0	29	1	1	0	1	1	0	2	0
1033	0	9	0	1	1	2	2	1	2	0
575	0	29	1	1	0	2	3	1	2	0
864	0	7	1	1	1	2	1	0	2	0
623	0	29	1	1	1	2	2	1	3	0
174	1	4	0	0	0	2	2	1	3	1
1244	0	29	1	0	0	1	1	0	2	0

1128	0	29	1	1	0	1	1	0	2	0
420	0	23	1	0	0	1	1	1	2	0
624	0	29	1	1	0	2	1	0	2	0
515	0	29	1	1	1	1	2	1	3	0
663	0	10	1	0	0	1	1	0	2	0
845	0	29	1	1	0	1	1	1	1	0
51	0	29	1	1	1	1	3	1	2	0
838	0	23	1	1	0	1	1	0	1	0
230	1	2	0	0	0	2	2	1	2	0
664	0	28	1	1	0	2	1	0	2	0
223	0	6	1	1	0	1	3	1	2	0
145	0	25	1	1	0	1	1	0	2	0
1101	1	5	1	1	0	2	3	1	3	1
808	1	1	0	0	0	2	3	1	2	1
318	0	29	1	1	0	1	2	0	3	0
419	0	13	1	1	0	1	2	0	2	0
899	0	29	0	1	1	1	1	0	2	0
143	0	28	1	1	0	2	1	0	2	0
783	0	26	1	0	1	1	2	1	3	0
542	0	29	0	1	0	1	1	0	2	0
1032	0	23	0	1	0	1	3	1	1	0
130	0	25	1	1	0	1	1	0	2	0
208	0	29	1	1	1	1	1	0	2	0
834	0	29	1	1	0	1	2	0	2	0
735	0	18	1	0	1	1	1	0	3	0
810	1	4	1	1	1	2	2	1	2	0
275	0	24	1	1	0	1	1	1	2	0
603	0	27	1	1	0	2	2	1	2	0
197	0	29	1	1	0	3	1	0	2	0
487	0	29	0	1	0	2	3	1	2	0
798	0	24	1	1	0	1	1	1	2	0
638	0	5	1	1	0	1	2	0	1	0
1232	0	27	1	1	0	1	1	0	2	0
1267	0	1	0	0	1	1	1	0	2	0
327	0	12	1	0	0	2	1	1	2	0
1000	0	28	0	0	1	1	2	1	3	1
895	1	4	1	0	1	2	1	0	2	1
1290	0	19	1	1	0	1	1	0	2	0

908	0	29	0	1	1	1	1	1	3	0
240	0	29	1	0	0	1	1	0	2	0
694	0	5	1	0	0	2	1	0	1	0
684	0	21	1	1	0	1	1	1	2	0
632	0	29	0	1	0	1	2	1	2	0
721	0	29	1	1	0	3	1	1	3	0
582	0	29	1	1	1	2	2	1	2	0
367	0	25	1	1	0	1	1	0	2	0
552	0	29	1	1	1	1	2	1	1	0
708	1	9	1	1	0	1	1	1	2	0
869	0	10	0	1	1	3	2	1	2	1
239	0	29	1	0	1	1	1	0	1	0
912	0	29	0	1	0	1	1	0	2	0
432	0	21	1	1	0	2	2	0	2	0
695	1	4	1	0	0	1	2	1	3	0
681	0	29	1	0	0	2	2	0	2	0
1253	0	6	0	0	0	2	3	0	2	0
906	0	29	0	1	1	2	1	0	2	0
998	0	29	0	0	1	1	1	0	1	0
1196	0	29	1	1	0	1	1	1	2	0
1051	0	29	0	0	1	2	1	0	2	0
405	0	27	1	1	0	1	1	0	2	0
1255	0	23	1	1	1	2	1	0	2	0
843	1	1	0	0	1	3	1	1	3	1
590	0	18	1	1	1	1	1	0	2	0
1301	0	19	0	1	1	2	1	0	2	0
943	0	29	1	0	0	1	1	0	2	0
1178	0	29	1	1	0	2	1	0	2	0
942	0	17	1	1	0	2	1	0	2	0
96	1	11	1	1	0	2	1	1	1	0
1276	0	29	0	1	0	2	3	1	2	0
467	0	29	1	1	0	1	2	1	2	0
1451	0	29	1	1	0	2	1	0	1	0
1184	0	24	1	0	0	1	1	1	2	0
345	0	29	1	1	0	2	2	0	2	0
659	0	11	0	1	0	2	1	1	2	0
909	0	18	0	1	0	1	1	1	2	0
109	0	23	1	0	0	1	1	1	2	1

382	0	29	1	1	0	2	2	0	3	0
596	0	29	1	1	1	1	2	1	2	0
733	0	29	1	1	0	1	1	0	2	0
159	0	29	1	0	0	1	1	0	2	0
1429	0	24	1	0	0	2	1	0	2	0
289	0	29	1	1	0	1	2	1	2	0
336	0	29	1	0	0	2	2	0	2	0
492	1	1	0	0	1	3	1	1	3	1
514	0	29	1	0	0	2	3	1	2	0
133	1	4	0	1	0	1	3	1	2	0
1374	0	26	1	0	0	2	1	0	2	0
202	0	29	1	1	0	1	1	0	1	0
955	0	27	1	1	0	2	1	0	2	0
621	0	29	1	0	0	2	1	1	2	0
1380	0	29	1	1	0	2	3	1	2	0
939	0	28	0	1	1	2	1	0	2	0
491	0	15	1	1	0	2	1	1	2	0
144	0	28	1	0	0	1	1	0	2	0
1061	0	22	0	1	1	1	2	1	3	0
252	0	29	1	0	0	3	1	0	2	1
607	0	29	1	0	1	1	1	0	2	0
865	0	24	0	0	0	1	1	0	2	0
89	0	20	1	1	1	1	2	1	2	0
462	0	7	1	1	0	1	2	1	2	0
1024	0	22	0	0	0	1	1	0	2	0
302	0	29	1	1	0	2	2	1	3	0
1439	0	18	1	1	0	3	1	1	2	0
961	0	29	1	0	0	2	1	0	2	0
1275	0	29	0	1	1	1	2	1	2	0
656	0	29	1	1	1	2	3	0	2	0
244	0	21	1	0	0	2	1	0	2	0
689	0	29	1	0	0	1	1	0	3	0
1067	0	29	0	1	0	2	1	0	2	0
142	0	29	1	1	0	1	1	0	2	0
868	0	15	1	1	1	2	2	0	2	0
1173	0	29	1	0	0	1	1	0	2	0
963	0	11	0	1	0	1	2	0	2	0
4	0	29	0	1	0	1	3	1	2	0

760	0	29	0	0	0	1	1	0	1	0
1240	0	18	1	0	0	2	3	0	3	0
210	0	29	1	1	0	1	1	1	1	0
1177	0	23	1	1	0	1	1	0	2	0
12	0	29	1	1	0	2	1	1	2	0
128	0	29	1	1	0	2	3	1	2	0
992	0	16	1	1	0	1	2	0	2	0
1142	0	28	0	0	0	1	1	0	2	0
800	0	29	1	0	0	1	1	0	2	0
1427	0	29	1	0	0	2	3	0	2	0
559	1	9	1	0	0	1	1	1	2	0
498	0	29	1	0	0	1	1	0	2	1
764	0	26	1	1	0	1	1	0	2	0
801	0	29	1	1	0	2	1	0	2	0
389	0	29	1	1	0	2	1	0	2	0
720	1	1	1	0	1	1	2	1	3	0
975	0	29	0	0	1	2	2	0	3	0
