# Supplementary material for: Prognostic model of HIV-associated talaromycosis in south China: A large-scale retrospective study
Source: PLoS Negl Trop Dis. 2025 Oct 30;19(10):e0013672. doi: 10.1371/journal.pntd.0013672 (PMC12591474; doi:10.1371/journal.pntd.0013672)
Supplement: S2 Data — (PDF) [file pntd.0013672.s002.pdf]

study_ID	Poor_outc	Time_disch	Poor_outc	Time_28D	Lymphade	Hepatospl	Tachypnea	WBC_strat	Thromboc	Severe_hy	LDH_strati	BUN_elevation
1	0	30	0	29	1	1	0	1	1	0	1	0
3	1	54	0	29	1	0	0	3	2	0	2	0
7	0	24	0	24	1	1	0	1	1	1	2	0
14	0	6	0	6	0	0	0	2	2	1	1	1
15	0	32	0	29	0	1	1	1	3	0	3	0
21	0	22	0	22	1	0	1	1	1	0	2	0
22	0	36	0	29	0	0	0	1	1	0	1	0
27	0	43	0	29	1	1	0	1	1	1	1	0
30	1	3	1	3	1	1	1	2	3	1	3	1
32	0	50	0	29	1	0	0	2	1	0	2	0
33	0	19	0	19	1	1	0	1	3	0	2	0
43	0	40	0	29	1	1	0	1	1	0	2	0
47	1	32	0	29	0	1	1	1	1	0	2	0
50	0	35	0	29	1	1	0	1	1	0	1	0
53	0	21	0	21	1	1	0	2	2	0	2	0
54	0	25	0	25	1	1	0	1	2	1	2	0
57	0	2	0	2	0	0	0	1	2	1	2	0
59	0	29	0	29	1	1	0	2	2	1	2	0
60	0	42	0	29	1	1	0	2	2	1	2	0
62	0	24	0	24	1	1	0	1	1	1	2	0
63	0	33	0	29	1	1	1	1	2	1	2	1
65	0	35	0	29	1	1	1	3	2	1	2	1
66	0	26	0	26	1	1	0	2	1	1	2	0
70	0	48	0	29	1	1	0	1	3	0	1	0
73	0	21	0	21	1	1	0	1	1	0	2	0
82	0	45	0	29	1	1	0	1	3	1	3	0
85	0	29	0	29	1	1	0	1	1	0	1	0
87	0	20	0	20	1	0	0	2	1	0	2	0
92	0	36	0	29	1	1	0	2	1	1	2	0
97	0	35	0	29	1	0	1	1	2	1	3	0
101	0	9	0	9	0	0	1	1	2	1	3	1
102	0	15	0	15	1	1	1	1	2	1	2	0
104	0	4	0	4	0	0	0	1	1	1	2	0
107	0	71	0	29	1	1	0	1	1	0	2	0
110	1	68	0	29	1	1	0	1	1	0	1	0
113	0	30	0	29	1	1	0	1	1	1	2	0
114	0	27	0	27	1	1	0	1	1	0	2	0

116	0	39	0	29	1	1	0	2	2	1	2	0
118	0	38	0	29	1	1	0	1	1	0	2	0
123	0	43	0	29	1	1	0	1	1	1	3	0
125	0	38	0	29	1	1	0	2	2	1	2	1
131	0	26	0	26	1	1	0	2	1	0	2	0
132	1	34	0	29	1	0	1	1	2	1	2	0
135	0	22	0	22	1	1	0	1	3	1	2	0
136	1	6	1	6	0	1	1	3	3	1	2	1
140	0	37	0	29	1	1	1	2	1	1	2	0
146	1	1	1	1	0	0	1	3	3	0	2	1
147	0	22	0	22	1	1	1	2	1	1	1	0
149	1	1	1	1	1	0	0	2	1	0	2	0
150	1	1	1	1	0	1	1	2	2	0	2	0
154	0	5	0	5	0	0	0	1	1	1	2	0
157	1	4	1	4	1	0	0	3	1	1	3	0
173	0	50	0	29	1	1	0	1	1	1	1	0
175	1	7	1	7	1	1	0	1	3	1	2	0
176	0	28	0	28	1	0	0	2	1	1	1	0
181	0	8	0	8	0	0	0	1	1	0	2	0
182	0	15	0	15	1	0	0	2	2	1	2	0
183	0	43	0	29	1	0	0	1	1	1	2	0
184	1	1	1	1	0	0	1	3	3	1	3	0
187	0	31	0	29	1	1	0	1	2	1	2	0
188	0	2	0	2	0	0	0	2	2	1	2	0
192	1	1	1	1	1	0	1	2	2	0	2	0
198	0	46	0	29	1	1	0	3	1	0	2	0
203	0	20	0	20	1	0	0	1	2	0	2	0
213	1	15	1	15	1	1	0	3	1	0	1	0
214	0	15	0	15	1	1	1	3	1	0	1	0
216	0	58	0	29	1	1	0	1	2	1	3	0
219	0	3	0	3	1	1	0	2	1	0	2	0
226	0	37	0	29	1	1	0	1	1	1	2	0
228	0	10	0	10	0	1	0	2	1	0	1	0
231	1	8	1	8	1	0	0	1	1	0	2	0
233	1	14	1	14	0	1	1	2	1	0	3	1
245	0	13	0	13	1	0	0	1	2	1	2	1
246	0	36	0	29	1	1	1	2	3	1	3	0
249	0	31	0	29	1	1	0	1	2	0	2	0

253	0	11	0	11	1	1	0	1	3	1	2	0
254	0	29	0	29	1	1	0	1	2	1	2	0
257	0	47	0	29	1	1	0	1	1	0	2	0
260	0	43	0	29	0	1	0	2	2	0	2	0
267	1	7	1	7	1	1	0	1	3	1	2	0
268	0	28	0	28	1	0	0	1	1	0	2	0
272	0	28	0	28	1	1	0	2	1	1	2	0
273	1	14	1	14	1	0	0	1	1	1	2	0
274	0	20	0	20	1	1	0	1	1	1	2	0
276	0	29	0	29	1	1	1	1	1	0	2	0
283	1	123	0	29	0	1	0	1	1	0	2	0
284	1	2	1	2	1	0	1	1	2	1	3	1
288	0	30	0	29	1	1	0	2	1	1	2	0
295	1	16	1	16	1	1	1	2	2	1	3	0
296	0	34	0	29	1	0	0	2	1	0	2	0
299	0	27	0	27	1	1	0	1	1	0	2	0
300	0	42	0	29	1	1	0	2	1	0	2	0
305	1	5	1	5	1	1	0	2	3	1	2	0
306	0	37	0	29	1	1	0	1	1	0	2	0
307	0	30	0	29	1	1	0	1	2	1	2	0
308	0	28	0	28	0	1	0	2	1	0	2	0
313	1	6	1	6	1	0	1	1	3	1	2	1
314	0	18	0	18	1	1	0	2	3	1	2	0
317	0	23	0	23	1	1	0	2	1	1	2	0
322	0	43	0	29	0	1	0	2	2	0	3	0
324	0	25	0	25	1	1	0	2	3	1	2	0
325	0	13	0	13	1	0	0	2	1	1	2	0
333	1	36	0	29	1	0	0	3	2	1	2	0
338	0	34	0	29	1	0	0	1	1	0	2	0
340	1	72	0	29	1	1	0	2	2	0	2	0
346	0	39	0	29	1	1	0	3	1	0	2	0
350	0	38	0	29	1	1	0	2	1	0	2	0
351	0	36	0	29	1	0	0	2	2	0	2	0
353	0	33	0	29	1	1	0	2	1	1	2	0
354	0	30	0	29	1	1	0	1	3	1	2	0
356	0	27	0	27	0	0	0	1	2	0	2	0
359	0	34	0	29	1	1	0	1	2	0	2	0
360	0	27	0	27	1	0	0	1	1	0	2	0

361	0	29	0	29	1	1	0	1	2	0	2	0
362	0	51	0	29	1	1	0	1	2	1	2	0
363	0	51	0	29	1	1	0	1	2	1	2	0
364	0	56	0	29	0	0	1	1	1	1	2	0
368	0	52	0	29	1	0	0	3	1	0	2	0
369	0	26	0	26	1	1	0	1	2	1	2	0
371	0	51	0	29	1	1	0	2	3	1	2	0
377	0	15	0	15	0	1	0	1	2	0	2	0
388	0	39	0	29	1	0	0	1	1	0	2	0
390	0	39	0	29	1	1	0	1	3	0	2	0
394	0	26	0	26	0	1	0	1	1	0	2	0
395	0	5	0	5	1	0	1	1	2	1	2	0
399	0	14	0	14	1	1	0	1	2	0	2	0
404	0	5	0	5	1	0	1	1	1	1	2	0
406	0	43	0	29	1	0	0	1	1	0	2	0
408	1	2	1	2	1	1	1	1	1	1	3	1
410	0	11	0	11	1	1	1	3	3	1	2	0
414	0	43	0	29	1	1	0	3	1	0	2	0
417	0	24	0	24	1	0	0	2	1	0	3	0
418	0	46	0	29	1	1	0	1	2	0	2	0
427	0	38	0	29	1	0	0	1	1	0	2	0
428	0	17	0	17	1	1	0	2	1	1	2	0
429	0	22	0	22	1	1	0	1	2	0	2	0
435	0	21	0	21	1	1	0	1	1	0	2	0
438	0	39	0	29	1	1	0	1	3	1	2	0
439	0	44	0	29	1	0	0	1	1	0	2	0
440	0	37	0	29	1	1	0	2	3	1	2	0
442	0	73	0	29	1	1	0	3	2	1	2	0
443	0	25	0	25	1	1	0	1	1	0	2	0
453	1	4	1	4	0	0	0	3	3	1	2	1
454	0	1	0	1	1	0	1	1	1	0	3	1
465	0	7	0	7	1	1	0	2	2	1	2	0
472	0	42	0	29	0	0	0	1	1	0	2	0
476	0	33	0	29	1	1	0	2	1	0	3	0
483	0	33	0	29	1	1	0	1	2	0	2	0
486	0	34	0	29	0	0	0	1	1	0	1	0
495	0	28	0	28	1	1	0	1	1	0	1	0
496	0	16	0	16	1	1	1	1	2	1	2	0

497	0	36	0	29	1	0	0	1	1	1	2	0
501	0	20	0	20	1	0	0	2	1	0	1	0
505	0	20	0	20	1	0	0	2	1	0	2	0
506	0	26	0	26	0	1	0	1	1	0	1	0
509	0	2	0	2	1	0	0	2	1	1	2	0
510	0	34	0	29	1	1	0	1	1	0	2	0
513	0	19	0	19	1	1	0	2	1	0	2	0
518	1	6	1	6	0	0	0	3	2	1	2	1
521	0	30	0	29	1	1	0	1	1	0	1	0
527	0	27	0	27	1	1	0	1	2	1	2	1
532	0	40	0	29	1	1	1	1	2	1	2	0
543	0	30	0	29	1	0	0	1	1	0	2	0
546	0	9	0	9	0	0	0	2	1	1	2	0
550	0	20	0	20	0	1	0	1	2	0	1	0
553	0	51	0	29	1	0	0	2	1	0	2	0
556	0	20	0	20	0	1	0	2	2	0	1	0
558	0	19	0	19	0	1	0	2	1	0	2	0
563	1	20	1	20	1	0	1	3	2	1	2	1
567	0	53	0	29	0	1	0	1	3	0	2	0
568	0	21	0	21	1	1	0	2	1	0	2	0
569	0	40	0	29	1	1	0	1	1	0	2	0
570	0	32	0	29	1	1	0	3	2	1	3	0
571	0	26	0	26	1	0	1	2	1	0	2	0
572	0	20	0	20	1	0	0	1	1	0	2	0
573	1	6	1	6	1	0	0	1	2	1	3	0
574	0	28	0	28	1	1	0	1	1	0	2	0
580	0	26	0	26	1	1	0	1	2	1	3	0
583	0	47	0	29	1	1	0	1	3	1	2	0
585	0	32	0	29	1	0	0	2	1	1	2	0
600	0	48	0	29	1	1	0	1	2	1	3	1
604	0	75	0	29	1	1	0	1	2	0	2	0
609	0	28	0	28	1	1	0	2	1	1	2	0
613	0	25	0	25	1	1	1	2	3	0	3	0
616	0	25	0	25	1	1	1	1	1	1	2	0
629	0	35	0	29	1	1	0	3	1	0	2	0
630	0	64	0	29	0	0	0	1	1	0	1	0
636	0	17	0	17	1	1	0	2	2	0	1	0
640	1	3	1	3	1	0	0	2	3	1	2	0

641	0	34	0	29	1	0	0	2	1	0	1	0
644	0	30	0	29	1	1	0	2	2	0	3	0
645	0	29	0	29	1	1	0	1	2	1	2	0
649	0	24	0	24	1	0	0	2	1	0	1	0
662	0	17	0	17	1	1	0	1	3	1	2	0
674	0	40	0	29	1	0	0	2	2	0	2	0
675	0	29	0	29	1	1	0	1	2	0	2	0
683	0	24	0	24	1	0	1	1	1	0	2	0
687	1	27	1	27	1	0	1	1	1	0	2	0
701	0	18	0	18	1	1	0	2	1	0	1	0
702	0	21	0	21	1	0	0	1	1	0	2	0
703	0	6	0	6	0	1	1	1	1	0	2	0
704	0	21	0	21	0	0	0	2	1	1	2	0
705	0	15	0	15	1	1	0	2	2	1	3	0
707	1	3	1	3	1	0	1	1	2	1	3	1
715	0	36	0	29	1	0	1	2	3	1	2	0
716	1	30	0	29	1	0	1	1	2	0	3	0
717	0	29	0	29	1	1	0	1	1	1	2	0
718	0	11	0	11	1	0	0	2	1	0	2	0
725	0	32	0	29	1	1	0	2	2	1	2	0
727	0	31	0	29	1	1	0	2	3	1	2	1
731	1	3	1	3	1	0	1	2	2	1	3	1
732	1	53	0	29	1	0	0	1	1	1	2	0
734	0	24	0	24	1	0	0	1	1	1	2	0
736	0	52	0	29	1	1	0	1	3	1	2	0
737	0	22	0	22	1	0	0	1	2	1	2	1
738	1	17	1	17	1	1	1	2	2	1	2	0
739	0	36	0	29	1	1	0	2	1	0	2	0
746	0	23	0	23	1	0	0	2	2	0	2	0
748	0	36	0	29	1	0	1	2	1	0	2	0
751	0	29	0	29	1	0	0	1	1	0	2	0
754	0	16	0	16	1	0	0	2	2	0	3	0
759	0	22	0	22	1	1	1	1	1	1	2	0
762	0	32	0	29	0	1	1	2	1	1	3	0
763	0	58	0	29	1	1	1	2	2	1	3	0
766	0	39	0	29	1	0	0	1	1	1	1	0
771	0	30	0	29	1	0	1	3	1	0	2	0
777	0	52	0	29	1	1	0	1	1	0	2	0

782	0	36	0	29	1	0	0	1	1	1	3	0
784	0	25	0	25	1	0	0	2	1	0	2	0
790	0	35	0	29	1	1	0	2	1	0	2	0
795	0	6	0	6	1	1	1	1	2	0	2	0
799	0	17	0	17	1	0	0	1	1	0	2	0
805	0	9	0	9	1	1	0	2	1	0	2	0
809	0	29	0	29	0	1	0	2	1	0	2	0
811	0	13	0	13	1	1	0	1	1	0	2	0
816	0	52	0	29	1	0	1	1	2	1	2	0
819	0	34	0	29	1	0	0	2	1	0	2	0
823	0	44	0	29	1	1	0	2	2	1	2	0
824	0	25	0	25	1	0	1	1	1	0	2	0
825	0	5	0	5	1	0	0	2	1	1	2	0
826	1	35	0	29	1	1	1	2	2	1	3	0
832	0	28	0	28	1	1	0	2	1	1	1	0
837	0	28	0	28	1	1	0	2	2	1	2	0
842	0	8	0	8	1	1	0	1	1	0	2	0
852	0	27	0	27	0	0	0	2	2	1	2	0
853	0	31	0	29	1	0	0	1	1	0	2	0
854	0	6	0	6	0	0	0	2	1	0	2	0
857	0	60	0	29	0	1	0	2	1	0	1	0
858	0	32	0	29	1	1	0	1	2	0	2	0
862	0	45	0	29	0	0	0	1	1	1	2	0
870	1	25	1	25	0	0	1	1	1	1	2	0
871	1	4	1	4	0	1	0	2	3	1	2	1
875	0	40	0	29	0	0	0	2	1	0	2	0
880	0	27	0	27	1	0	0	2	1	1	2	0
881	0	16	0	16	0	1	0	2	2	1	1	1
882	0	26	0	26	0	1	1	2	1	0	2	0
885	0	50	0	29	0	1	1	2	3	1	3	0
888	0	27	0	27	0	0	1	1	1	0	2	0
892	0	34	0	29	1	1	1	1	2	1	2	0
893	0	25	0	25	0	0	0	2	1	0	2	0
894	0	25	0	25	1	1	0	2	1	0	1	0
900	1	6	1	6	0	0	1	2	3	1	2	1
901	0	11	0	11	0	1	1	3	1	1	2	0
907	0	37	0	29	1	1	0	2	3	1	3	1
910	0	33	0	29	0	0	0	1	1	0	2	0

911	0	32	0	29	0	0	0	2	2	1	3	0
913	0	30	0	29	0	1	0	1	1	0	2	0
914	0	30	0	29	0	1	1	2	2	1	3	0
915	0	26	0	26	0	1	0	3	2	1	3	0
917	0	26	0	26	0	1	1	1	2	0	2	0
922	0	29	0	29	1	1	1	2	1	1	2	0
934	0	36	0	29	1	1	0	1	2	1	2	0
936	0	26	0	26	0	1	0	1	1	1	2	0
940	0	35	0	29	0	1	0	2	2	0	2	0
949	0	18	0	18	0	1	0	1	2	1	3	0
952	0	9	0	9	0	1	1	1	1	0	2	0
954	0	46	0	29	0	1	0	3	2	1	2	0
958	0	38	0	29	0	0	0	1	2	1	2	0
962	0	34	0	29	0	1	0	3	2	1	2	0
964	1	12	1	12	0	0	1	3	2	0	3	0
965	0	19	0	19	0	1	1	1	1	0	2	0
966	0	35	0	29	0	0	0	1	2	0	1	0
967	0	32	0	29	1	1	0	1	2	0	3	0
969	0	31	0	29	1	1	0	1	2	1	2	0
970	0	36	0	29	1	1	0	1	1	0	3	0
977	0	49	0	29	0	0	1	2	2	1	2	0
979	0	12	0	12	0	1	0	1	2	1	2	0
982	1	3	1	3	1	0	0	3	1	1	3	1
984	0	16	0	16	0	1	0	1	1	0	2	0
1001	1	4	1	4	0	0	1	1	2	1	3	1
1003	0	20	0	20	1	1	1	2	2	1	2	0
1007	0	24	0	24	0	0	1	1	2	0	2	0
1008	0	7	0	7	0	0	0	1	1	0	2	0
1009	0	37	0	29	0	1	0	1	1	0	1	0
1010	0	23	0	23	0	0	0	1	2	1	3	0
1012	0	28	0	28	1	1	0	1	2	0	2	0
1015	0	30	0	29	0	1	1	1	1	1	3	0
1019	0	26	0	26	1	1	1	1	1	0	2	0
1020	0	28	0	28	1	0	0	1	1	1	2	0
1022	1	27	1	27	0	1	1	1	1	1	2	0
1025	0	9	0	9	1	1	0	1	1	0	2	0
1026	0	31	0	29	0	1	0	1	2	1	2	0
1035	0	12	0	12	0	1	0	2	1	1	2	0

1037	0	26	0	26	1	1	0	1	1	0	2	0
1039	0	3	0	3	0	0	0	2	1	0	2	0
1040	0	28	0	28	0	1	0	1	1	1	1	0
1045	0	16	0	16	0	0	0	2	1	1	2	1
1049	0	22	0	22	0	1	0	1	2	0	3	0
1050	0	23	0	23	0	1	0	1	1	1	1	0
1052	0	63	0	29	0	0	0	1	1	1	2	0
1053	0	47	0	29	0	0	1	3	2	1	3	0
1058	0	9	0	9	0	1	0	1	1	1	2	0
1064	0	22	0	22	0	1	0	1	2	1	2	0
1066	0	31	0	29	0	1	0	2	2	1	3	0
1071	1	35	0	29	0	1	1	2	2	1	3	0
1073	0	21	0	21	0	0	0	1	2	0	2	0
1077	0	27	0	27	0	1	0	2	1	1	2	0
1080	0	35	0	29	1	1	0	2	2	1	1	0
1089	0	38	0	29	1	0	0	1	1	0	1	0
1092	0	21	0	21	1	1	0	3	1	0	2	1
1096	0	7	0	7	0	0	0	1	1	0	1	0
1107	0	4	0	4	1	0	0	2	1	0	2	0
1117	0	24	0	24	1	1	0	1	2	0	3	0
1119	0	40	0	29	0	1	0	1	2	0	2	0
1122	1	9	1	9	0	1	0	2	2	1	2	1
1123	0	22	0	22	1	1	0	1	2	0	3	0
1125	0	2	0	2	1	0	0	2	2	1	2	0
1132	1	4	1	4	0	1	0	3	2	1	2	0
1136	0	26	0	26	1	1	0	1	1	1	2	0
1137	0	63	0	29	0	0	0	1	1	0	2	1
1139	0	34	0	29	1	0	0	1	1	0	2	0
1141	0	16	0	16	0	0	0	3	3	1	2	1
1146	0	39	0	29	1	1	0	3	2	1	2	0
1149	0	1	0	1	0	0	0	1	2	0	2	0
1150	0	31	0	29	0	0	0	2	1	0	3	0
1152	0	35	0	29	1	1	0	1	1	0	2	0
1153	0	27	0	27	1	1	0	1	1	0	2	0
1154	0	19	0	19	1	0	0	2	1	0	2	1
1157	0	24	0	24	1	0	0	2	1	0	2	0
1159	0	50	0	29	1	1	0	1	2	0	1	0
1160	0	21	0	21	1	0	0	1	1	1	2	0

1165	0	21	0	21	1	1	0	1	1	0	2	0
1166	0	17	0	17	1	0	0	2	1	0	2	0
1168	0	37	0	29	1	0	0	2	2	0	2	0
1172	0	39	0	29	1	0	0	2	1	1	1	0
1182	1	7	1	7	1	0	0	2	2	0	2	1
1185	0	54	0	29	1	1	0	1	2	0	2	0
1187	0	24	0	24	1	1	1	1	2	0	2	0
1189	0	22	0	22	1	1	0	1	1	0	2	0
1192	0	31	0	29	1	0	0	2	1	0	1	0
1199	0	29	0	29	1	1	0	1	1	0	1	0
1201	0	23	0	23	1	0	1	1	1	0	2	0
1206	0	12	0	12	1	1	1	2	3	1	2	0
1208	0	20	0	20	1	1	1	3	3	1	2	1
1211	1	38	0	29	0	0	0	2	2	0	2	0
1213	0	52	0	29	1	1	0	2	2	0	2	0
1219	0	15	0	15	0	0	1	2	1	0	2	0
1221	0	24	0	24	1	1	0	2	1	0	2	0
1222	0	29	0	29	0	0	0	2	2	0	2	0
1226	0	26	0	26	1	1	0	2	1	1	1	0
1227	0	31	0	29	1	0	1	1	1	0	2	0
1228	0	35	0	29	1	0	1	1	2	1	2	1
1229	0	26	0	26	1	1	0	1	1	0	2	0
1230	0	33	0	29	1	1	0	2	1	0	2	0
1233	0	46	0	29	1	1	0	1	1	0	2	0
1237	0	24	0	24	1	1	0	2	2	0	2	0
1239	0	19	0	19	1	1	0	1	2	0	1	0
1245	1	15	1	15	0	0	0	1	2	1	2	1
1254	0	29	0	29	1	0	1	2	1	1	2	0
1265	0	24	0	24	1	1	0	2	2	1	2	0
1271	0	27	0	27	1	1	0	2	2	0	2	0
1277	0	16	0	16	1	1	0	1	2	1	3	0
1278	0	34	0	29	0	1	0	2	1	0	2	0
1280	0	47	0	29	1	1	1	1	3	1	2	0
1281	0	30	0	29	0	1	0	1	2	0	2	0
1282	0	43	0	29	1	1	0	3	1	0	1	0
1284	0	32	0	29	1	1	0	1	1	0	2	0
1289	0	33	0	29	1	1	0	1	1	0	2	1
1295	0	36	0	29	1	1	0	3	2	0	3	0

1297	0	42	0	29	1	1	0	1	1	0	2	0
1305	1	3	1	3	1	1	0	1	2	1	3	1
1307	0	45	0	29	1	1	0	1	1	0	2	0
1311	0	12	0	12	1	0	0	1	1	0	2	0
1320	0	28	0	28	1	0	0	1	1	0	2	0
1322	0	24	0	24	1	1	0	1	1	0	1	0
1325	0	40	0	29	1	0	0	2	2	1	2	1
1331	0	28	0	28	0	0	0	2	1	0	2	0
1337	0	51	0	29	1	0	0	1	1	0	1	0
1339	0	20	0	20	1	1	0	2	2	0	2	0
1340	0	15	0	15	1	0	0	1	1	0	2	0
1343	0	16	0	16	1	0	0	1	1	0	2	1
1347	0	27	0	27	1	0	0	2	1	0	2	0
1349	1	17	1	17	1	0	0	2	3	1	1	0
1352	0	26	0	26	1	1	0	1	1	0	2	0
1353	0	23	0	23	1	0	0	1	1	0	2	0
1358	1	7	1	7	1	0	0	2	3	1	3	0
1359	0	63	0	29	1	1	1	1	2	1	3	0
1365	1	1	1	1	1	0	1	1	1	1	2	0
1367	0	44	0	29	1	1	1	1	2	0	2	0
1371	0	29	0	29	1	0	0	2	1	1	1	0
1376	1	6	1	6	0	0	0	1	2	1	2	1
1381	0	6	0	6	1	1	0	2	2	1	2	1
1382	0	3	0	3	1	1	0	2	1	0	2	0
1384	0	11	0	11	1	0	1	1	2	1	2	0
1389	0	21	0	21	1	1	0	3	2	1	3	0
1391	0	46	0	29	0	1	0	2	1	0	1	0
1393	0	49	0	29	0	1	1	2	1	1	2	0
1398	0	3	0	3	1	0	0	2	1	0	1	0
1403	0	31	0	29	1	0	0	2	1	0	2	0
1404	0	4	0	4	1	1	0	1	2	1	2	0
1409	0	29	0	29	1	0	0	2	1	0	2	0
1414	0	14	0	14	0	0	0	1	1	0	1	0
1415	1	5	1	5	1	0	0	1	2	0	1	0
1419	1	1	1	1	1	0	0	1	3	0	2	0
1421	1	6	1	6	0	0	0	2	2	1	2	1
1424	0	15	0	15	1	1	0	1	1	0	2	0
1426	0	19	0	19	0	0	0	2	1	0	2	0

1432	0	19	0	19	1	0	0	2	1	0	1	0
1434	0	6	0	6	0	1	1	3	2	0	3	0
1435	0	20	0	20	1	0	0	2	1	1	2	0
1440	0	5	0	5	1	1	0	2	2	0	2	0
1444	0	22	0	22	0	0	0	1	1	0	2	1
1446	0	32	0	29	1	1	0	1	1	1	2	0
1448	0	52	0	29	1	1	0	1	2	0	2	0
1455	0	85	0	29	1	1	0	1	1	0	2	0
1456	0	26	0	26	1	0	1	1	1	0	2	0
1458	0	44	0	29	1	1	0	2	2	1	2	0
1466	0	32	0	29	1	1	0	2	2	0	2	0
1467	0	7	0	7	0	0	0	1	1	0	2	0
1472	0	38	0	29	1	0	0	2	1	1	2	0
1473	0	26	0	26	1	0	0	1	1	0	1	0
