# Supplementary material for: Prognostic model of HIV-associated talaromycosis in south China: A large-scale retrospective study
Source: PLoS Negl Trop Dis. 2025 Oct 30;19(10):e0013672. doi: 10.1371/journal.pntd.0013672 (PMC12591474; doi:10.1371/journal.pntd.0013672)
Supplement: S3 Data — (PDF) [file pntd.0013672.s003.pdf]

study_ID	Poor_outc	Time_28D	Lymphade	Hepatospl	Tachypnea	WBC_strat	Thromboc	Severe_hy	LDH_strati	BUN_elevation
1081	0	26	0	1	0	2	2	0	2	0
1082	0	25	1	1	0	2	2	1	2	0
1084	1	5	0	0	0	1	1	0	2	1
1085	1	7	0	0	0	3	2	1	3	0
1086	0	17	1	0	0	2	1	0	2	0
1087	1	13	1	1	0	1	1	0	2	0
1090	0	27	1	1	0	2	2	0	2	0
1091	1	3	0	0	1	1	2	0	2	1
1093	1	2	0	1	1	2	2	1	3	1
1094	0	29	0	0	0	2	1	0	2	0
1095	0	18	1	0	0	1	1	0	2	1
1097	0	19	0	1	0	1	2	0	1	0
1098	0	22	1	1	0	1	1	0	2	0
1099	0	29	1	0	0	2	1	0	2	0
1100	0	28	1	1	0	1	1	0	1	0
1105	1	7	0	0	0	1	2	0	2	1
1106	0	27	0	0	0	2	1	0	2	0
1108	1	5	1	0	0	1	2	1	1	0
1110	0	29	0	0	0	2	2	1	2	0
1111	0	27	0	0	0	1	1	0	2	0
1151	0	19	0	0	1	1	2	0	2	0
1225	0	10	1	0	1	1	2	0	2	0
1231	0	22	0	1	0	1	3	1	2	0
1247	0	17	0	0	1	2	1	0	2	0
1262	0	29	0	0	0	1	2	1	2	0
1269	0	29	0	0	0	2	2	1	1	0
1293	0	15	0	0	0	1	2	0	3	0
1294	0	10	0	0	0	1	1	0	2	0
1304	0	29	0	0	0	1	1	0	1	0
1316	0	16	1	1	0	2	2	0	2	0
1318	0	11	1	1	0	2	1	0	2	0
1348	0	27	0	0	0	1	1	0	1	1
1370	0	29	0	0	1	1	1	1	1	1
1383	0	29	1	1	1	2	1	1	2	0
1418	0	28	1	0	1	2	2	1	3	0
1438	0	14	1	1	0	2	2	0	3	0
1452	0	22	1	0	0	1	1	0	1	0

1457	0	4	1	1	0	1	1	0	1	0
1462	0	8	0	1	0	1	1	0	2	0
1468	1	9	0	0	0	1	1	1	2	1
1474	0	11	0	1	0	1	2	1	2	0
1477	0	29	0	0	0	1	2	1	2	0
1478	1	1	0	0	1	2	3	0	2	1
1479	0	20	0	0	0	1	1	0	2	0
1480	0	29	1	1	1	2	1	0	1	1
1481	1	22	0	1	1	1	3	1	2	0
1482	0	18	1	1	0	2	1	0	2	0
1483	1	2	0	0	1	3	2	1	3	0
1484	0	29	1	1	1	2	2	1	2	1
1485	0	18	0	0	0	1	1	0	2	1
1486	0	17	0	1	0	1	1	0	2	0
1487	1	23	1	1	1	1	2	1	1	0
1488	0	29	0	0	0	2	1	0	2	0
1489	0	22	0	0	0	2	1	0	1	0
1490	0	29	1	1	0	2	1	1	2	0
1491	0	25	0	0	1	3	1	1	2	0
1492	1	1	0	0	1	1	2	0	3	1
1493	0	6	1	0	0	1	1	1	2	1
1494	0	14	1	1	0	3	2	0	1	0
1495	0	20	1	1	0	2	1	0	2	0
1496	1	1	1	0	1	3	3	1	3	1
1497	0	11	0	0	1	1	1	1	2	0
1498	1	2	0	1	1	2	2	0	3	1
1499	1	3	1	1	1	2	1	1	2	1
1500	0	19	0	0	0	1	1	0	2	0
1501	0	28	1	0	0	1	1	0	2	0
1502	0	29	1	1	1	3	2	1	2	0
1503	0	29	1	0	0	3	1	0	1	0
1504	0	5	1	1	0	1	1	1	2	0
1505	0	29	0	1	0	2	2	1	2	0
1506	1	4	0	0	0	2	3	1	3	1
1507	1	5	0	0	1	2	2	1	3	1
1508	0	29	1	1	0	3	2	1	2	0
1509	0	5	1	1	0	2	2	1	2	0
1510	0	5	0	0	0	1	1	0	1	0

1511	0	6	1	1	0	1	3	1	2	1
1512	0	9	1	0	0	3	1	1	2	0
1513	0	29	1	0	0	1	1	0	2	0
1514	0	29	1	0	1	1	1	0	2	0
1515	0	18	1	0	0	1	1	0	2	0
1516	0	26	1	0	1	1	1	0	1	0
1517	0	19	0	0	1	1	1	0	2	0
1518	0	29	0	0	0	1	1	0	1	0
1519	0	6	0	0	1	1	1	0	1	0
1520	0	29	1	1	0	1	2	1	2	0
1521	0	14	1	0	0	1	1	0	2	0
1522	0	29	1	1	0	1	3	1	1	0
1523	0	27	1	0	0	2	1	0	2	0
1524	0	29	0	0	0	2	1	0	2	0
1525	1	3	0	0	0	1	2	1	3	0
1526	0	29	1	1	0	2	1	1	2	0
1527	0	3	0	1	0	2	1	0	2	0
1528	0	29	1	0	0	1	2	0	2	0
1529	0	23	1	0	0	1	1	0	2	0
1530	0	3	1	1	0	2	1	0	1	0
1531	0	29	0	1	0	1	1	1	2	0
1532	0	29	1	1	0	2	2	0	2	0
1533	0	24	1	0	0	1	1	0	2	0
1534	1	3	1	1	0	1	2	1	2	0
1535	0	29	1	0	1	1	1	0	2	0
1536	1	2	0	0	1	1	3	1	3	1
1537	0	21	1	0	0	1	1	0	2	0
1538	0	29	1	1	0	1	1	0	2	0
1539	0	29	1	1	0	1	1	0	2	0
1540	0	22	1	0	0	3	1	0	1	0
1541	0	15	1	0	0	1	1	1	2	0
1542	0	29	1	1	0	1	1	0	2	0
1543	0	18	1	0	0	1	1	1	2	0
1544	1	3	0	0	1	3	3	1	3	1
1545	1	3	1	1	0	1	2	1	2	0
1546	0	3	1	1	0	2	2	1	1	0
1547	0	16	1	0	0	2	2	1	2	1
1548	0	18	1	1	0	2	3	0	2	1

1549	0	4	0	0	0	1	3	1	3	1
1550	0	29	1	1	0	2	2	0	2	0
1551	0	12	1	1	0	1	1	0	2	0
1552	1	2	0	0	1	1	2	1	3	0
1553	1	6	1	1	0	3	2	1	3	1
1554	0	7	1	0	0	1	1	0	2	0
1555	0	14	1	1	0	2	3	1	2	0
1556	0	25	0	1	0	1	1	1	1	0
1557	0	29	1	1	0	1	2	1	2	0
1558	0	29	1	1	1	1	2	0	2	0
1559	0	29	1	0	0	1	1	1	2	0
1560	0	12	1	0	0	1	1	0	2	0
1561	0	29	0	0	1	1	1	0	2	0
1562	0	12	1	1	0	2	1	0	1	0
1563	0	29	0	0	1	1	1	0	2	0
1564	0	29	1	1	0	1	3	0	2	1
1565	1	27	0	0	0	1	1	0	2	1
1566	0	8	1	0	0	2	2	0	2	0
1567	1	2	0	1	1	1	2	1	2	0
1568	0	16	0	1	1	3	2	0	2	0
1569	0	26	1	1	0	1	3	1	2	1
1570	0	14	1	0	0	2	1	0	2	0
1571	0	16	0	1	0	1	1	0	2	0
1572	0	11	0	0	0	1	1	0	1	0
1573	1	21	0	1	1	1	1	1	2	0
1574	0	28	1	1	1	2	2	0	2	0
1575	0	29	1	0	1	2	3	0	3	0
1576	0	29	1	0	1	1	1	0	2	0
1577	0	29	1	1	0	1	1	0	1	0
1578	0	4	0	1	0	1	1	0	2	0
1579	1	8	1	1	0	2	2	1	2	0
1580	1	9	0	0	1	1	1	0	2	0
1581	0	29	1	0	0	1	1	1	2	0
1582	0	29	0	1	0	2	2	1	2	0
1583	0	19	1	1	0	2	1	0	1	0
1584	1	4	1	1	0	1	3	1	2	0
1585	0	29	0	0	0	1	1	0	1	0
1586	0	21	1	1	0	2	1	0	2	0

1587	1	4	1	0	1	1	3	1	3	1
1588	0	29	1	1	0	2	2	1	2	0
1589	0	29	1	1	0	1	2	1	2	0
1590	0	10	1	1	1	1	2	0	2	1
1591	0	29	1	1	0	1	1	0	2	0
1592	0	10	1	0	0	1	2	0	2	0
1593	0	11	1	0	0	1	1	1	2	0
1594	0	29	1	0	1	3	2	0	3	0
1595	0	29	1	0	1	1	1	0	1	0
1596	0	29	0	1	1	2	1	0	1	0
1597	0	4	0	1	0	1	1	0	1	0
1598	0	18	0	0	1	2	1	1	2	0
1599	0	29	0	0	1	3	1	0	2	1
1600	0	29	1	1	0	1	3	0	1	0
1601	0	29	1	1	0	1	3	1	2	0
1602	0	29	0	0	0	2	2	0	2	0
1603	0	29	0	1	0	1	1	0	2	0
1604	0	28	0	1	0	2	1	1	2	0
1605	0	29	1	1	0	2	2	1	2	0
1606	0	25	1	1	0	1	2	0	3	0
1607	1	16	1	1	0	1	2	1	2	1
1608	0	29	1	1	0	1	2	0	2	0
1609	0	29	1	1	0	2	3	0	2	0
1610	0	29	1	1	0	2	2	1	3	0
1611	0	6	0	1	1	2	1	0	2	0
1612	0	29	1	1	0	1	1	0	2	0
1613	0	29	1	0	1	1	1	0	2	0
1614	0	29	1	1	0	1	1	1	2	0
1615	1	9	0	0	1	2	2	0	2	0
1616	0	29	1	1	0	2	1	0	1	0
1617	0	29	0	0	0	2	1	0	2	0
1618	0	27	0	0	1	2	1	0	2	0
1619	0	24	1	1	0	2	2	1	2	0
1620	0	2	1	0	0	1	1	0	2	1
1621	0	29	1	1	0	2	3	0	2	1
1622	0	25	0	0	0	1	1	0	1	0
1623	0	29	1	1	1	1	2	0	1	0
1624	0	16	1	1	0	1	1	0	2	0

1625	0	29	1	1	0	1	1	1	2	0
1626	0	29	1	0	0	2	2	1	2	0
1627	0	29	0	1	0	2	2	0	2	0
1628	0	29	1	1	1	2	1	0	2	0
1629	0	29	1	1	0	2	2	0	2	0
1630	0	11	1	1	0	2	2	0	2	1
1631	0	29	0	0	0	2	2	1	2	0
1632	0	29	1	0	0	1	1	0	2	0
1633	0	29	1	0	0	1	1	1	2	0
1634	0	29	0	1	0	1	1	0	2	0
1635	0	5	1	1	0	1	1	1	1	0
1636	0	29	1	1	1	1	3	1	2	0
1637	0	29	1	1	0	1	2	0	2	0
1638	0	29	1	0	0	1	1	0	2	0
1639	1	13	0	0	0	2	1	1	2	1
1640	0	29	1	1	1	1	2	1	2	1
1641	1	5	0	0	1	1	1	1	2	1
1642	1	7	0	1	0	1	3	1	3	1
1643	0	12	0	0	0	1	1	0	2	0
1644	0	29	0	1	0	2	1	1	2	0
1645	1	5	1	1	0	2	1	1	2	1
1646	0	29	1	0	0	2	1	0	1	0
1647	0	29	0	1	1	1	1	0	2	1
1648	0	8	0	0	0	1	1	0	2	0
1649	0	29	0	1	0	2	1	0	2	0
1650	0	29	1	1	0	2	2	0	2	1
1651	0	16	0	0	0	2	1	0	2	0
1652	0	5	1	1	0	1	2	0	2	0
1653	0	29	1	1	1	3	2	1	2	0
1654	0	29	1	1	0	1	2	0	2	0
1655	0	29	1	1	0	2	3	1	3	1
1656	0	29	1	0	0	2	2	0	2	0
1657	0	21	0	0	0	1	1	0	1	0
1658	1	25	0	0	0	1	2	1	2	0
1659	0	29	0	0	1	1	2	1	1	1
1660	0	29	0	1	0	2	1	0	2	1
1661	0	29	1	1	0	3	2	1	2	0
1662	0	23	1	0	0	1	1	0	1	0

1663	0	29	1	0	0	2	1	0	1	0
1664	0	29	1	1	0	2	2	0	2	0
1665	0	24	1	0	1	1	1	0	2	0
1666	0	29	1	0	1	1	1	0	2	0
1667	0	28	0	1	0	2	2	0	3	0
1668	0	22	1	1	0	1	3	0	2	0
1669	0	29	1	0	0	2	2	0	2	0
1670	0	29	1	1	0	2	1	0	2	0
1671	0	6	1	1	0	2	2	1	2	0
1672	0	29	1	0	0	2	1	0	2	0
1673	0	5	1	1	0	2	1	0	2	0
1674	1	3	0	1	1	2	2	1	3	0
1675	0	21	1	0	0	2	2	0	2	0
1676	0	29	0	1	0	1	1	0	1	0
1677	0	21	1	1	1	1	1	1	2	0
1678	0	22	0	0	1	2	3	1	2	0
1679	0	29	0	1	1	1	1	0	2	0
1680	0	27	1	1	0	1	2	1	2	1
1681	0	17	1	1	0	2	2	1	2	0
1682	0	27	0	1	1	1	2	0	3	1
1683	0	2	1	0	0	1	2	0	2	0
1684	0	9	0	1	0	2	1	1	2	0
1685	0	5	0	1	0	1	2	0	2	0
1686	0	29	0	1	1	2	1	0	2	0
1687	0	29	1	1	1	1	1	0	2	0
1688	0	28	1	0	0	1	1	0	1	0
1689	0	22	1	1	0	1	1	0	2	0
1690	0	28	0	0	0	2	1	0	1	0
1691	0	8	0	0	1	2	1	1	2	0
1692	0	29	0	1	0	1	2	1	2	0
1693	0	29	1	1	0	2	3	0	2	0
1694	0	24	0	0	0	1	2	0	2	0
1695	0	29	1	1	1	2	2	1	2	0
1696	0	19	0	0	0	1	1	0	2	0
1697	0	21	1	1	0	1	3	1	2	0
1698	1	16	0	1	0	1	3	1	3	1
1699	0	22	0	0	0	1	1	1	2	0
1700	0	20	1	1	0	2	2	0	2	0

1701	0	18	0	0	0	2	2	0	1	1
1702	0	24	1	0	0	2	2	1	2	1
1703	0	29	0	1	0	1	1	1	1	0
1704	0	29	0	1	1	1	3	0	2	0
1705	0	27	1	1	0	1	1	1	2	0
1706	0	29	0	1	0	1	3	0	2	0
1707	0	29	1	1	1	1	1	1	2	0
1708	0	29	1	0	0	1	1	0	3	0
1709	0	25	1	0	0	2	1	0	2	0
1710	0	29	0	1	0	2	2	1	2	0
1711	1	3	0	1	1	3	2	1	3	1
1712	0	29	1	1	0	2	2	1	2	0
1713	1	26	1	0	0	1	1	0	2	0
1714	0	29	0	0	1	3	1	0	2	1
1715	0	23	0	0	0	3	1	0	2	0
1716	0	28	0	1	0	1	1	0	1	0
1717	0	29	0	0	0	1	1	0	2	0
1718	0	20	1	0	0	1	2	0	2	0
1719	0	26	1	1	0	2	2	1	3	0
1720	0	29	1	0	0	2	2	0	1	0
1721	0	29	0	0	1	1	1	0	2	0
1722	1	1	0	0	0	3	1	0	2	0
1723	0	27	0	0	0	2	1	1	1	0
1724	0	19	0	0	0	1	1	0	1	0
1725	0	29	0	1	0	1	1	0	1	0
1726	1	1	0	0	1	3	3	1	3	0
1727	0	18	1	0	0	1	1	0	1	0
1728	0	29	1	0	0	2	1	0	2	0
1729	0	6	1	1	0	1	1	0	2	0
1730	0	7	0	1	1	3	3	1	3	1
1731	0	17	0	1	0	1	1	0	1	0
1732	0	18	1	1	1	1	2	1	2	0
1733	0	29	1	0	1	1	2	1	3	0
1734	0	15	0	1	1	2	1	0	2	0
1735	0	23	1	0	0	2	1	0	1	0
1736	0	29	1	0	0	2	1	0	1	0
1737	0	29	1	0	1	1	3	0	1	0
1738	0	29	1	1	0	2	2	0	2	0

1739	0	29	1	0	1	1	1	1	2	0
1740	0	29	1	0	0	1	1	0	2	0
1741	0	29	0	0	0	2	1	0	1	0
1742	0	29	0	0	0	1	2	0	2	0
1743	0	21	0	0	0	1	1	0	1	0
1744	0	13	1	1	0	1	3	0	2	0
1745	1	28	0	1	0	2	2	1	2	1
1746	0	29	1	0	0	1	1	0	1	0
1747	0	29	1	1	0	1	1	0	1	0
1748	0	24	0	1	0	1	1	0	1	0
1749	0	29	1	1	0	2	1	0	1	0
1750	0	27	0	1	0	2	2	0	2	0
1751	1	28	0	0	0	2	2	1	2	0
1752	0	17	0	0	0	2	2	0	2	0
1753	0	29	1	0	0	1	1	1	1	0
1754	0	23	1	1	0	1	1	1	2	0
1755	1	2	0	0	0	1	2	1	3	1
1756	0	29	1	0	0	2	2	1	2	1
1757	0	29	1	0	0	1	1	0	1	0
1758	0	8	0	0	0	1	1	0	1	0
1759	0	29	1	1	0	1	3	1	2	1
1760	0	29	0	1	0	2	2	0	2	0
1761	0	29	1	1	1	1	1	0	2	0
1762	0	27	0	0	0	1	1	0	1	0
1763	0	29	1	0	1	1	3	1	1	1
1764	1	3	0	1	1	1	2	1	3	1
1765	1	5	1	0	0	2	3	0	2	1
1766	0	29	0	0	0	2	1	0	2	0
1767	0	29	0	0	1	1	1	1	2	0
1768	0	29	1	1	0	1	1	0	2	0
1769	0	29	0	0	1	1	1	0	1	0
1770	0	29	0	0	0	1	1	0	2	0
1771	1	12	0	0	0	2	3	1	2	0
1772	0	29	1	1	0	2	2	1	2	0
1773	0	29	0	0	1	1	1	0	2	0
1774	0	29	0	0	1	1	1	0	2	0
1775	0	29	1	1	0	1	1	0	2	0
1776	0	18	1	0	0	1	1	0	2	0

1777	0	25	0	0	0	1	1	1	1	0
1778	0	29	1	1	0	1	1	0	2	0
1779	0	29	0	0	0	3	1	1	1	0
1780	0	29	0	1	0	3	1	1	2	0
1781	0	29	0	0	0	1	1	0	2	0
1782	0	29	1	1	0	1	2	0	2	0
1783	0	29	0	0	0	1	1	0	2	0
1784	0	26	1	1	0	1	1	0	2	0
1785	0	23	1	1	1	1	1	0	1	1
1786	0	29	0	1	1	2	2	1	2	0
1787	0	22	0	1	1	1	2	0	2	0
1788	0	29	1	1	0	1	2	0	3	1
1789	0	23	1	0	1	1	2	0	2	0
1790	0	29	1	1	1	1	2	1	2	0
1791	1	3	0	1	1	3	2	1	3	0
1792	0	29	0	0	1	2	2	0	2	0
1793	0	29	1	1	0	1	2	0	2	0
1794	1	15	0	0	1	1	1	1	2	0
1795	0	29	1	1	0	1	2	0	2	0
1796	0	29	1	1	0	2	1	0	2	0
1797	0	29	0	0	0	1	1	0	1	0
1798	0	29	1	1	1	1	2	0	3	0
1799	0	27	1	1	0	3	2	1	2	0
1800	0	22	0	0	0	1	2	0	2	0
1801	0	29	1	1	1	1	1	1	2	0
1802	0	29	0	0	0	1	1	0	1	0
1803	0	16	1	0	0	1	1	0	2	0
1804	0	29	1	1	0	1	2	1	2	0
1805	0	29	0	0	0	1	1	0	2	0
1806	0	12	1	1	0	1	3	0	3	1
1807	0	21	1	1	1	1	2	0	2	0
1808	0	29	1	1	1	1	1	0	2	0
1809	0	16	0	0	0	1	2	0	2	1
1810	0	29	1	1	0	2	2	1	2	0
1811	1	2	0	1	0	1	2	0	3	0
1812	0	29	1	0	0	1	1	0	2	0
1813	0	29	1	1	0	1	1	0	2	0
1814	0	28	1	1	0	1	3	1	2	0

1815	0	29	1	0	0	1	1	0	1	0
1816	0	29	1	1	0	1	2	1	2	0
1817	0	28	1	0	0	2	1	0	2	0
1818	1	2	0	0	0	1	1	0	2	0
1819	0	29	0	0	1	1	1	0	2	0
1820	0	24	1	1	0	2	2	1	3	1
1821	1	11	1	1	1	2	3	1	2	1
1822	0	29	1	0	0	1	1	0	2	0
1823	0	29	1	1	0	2	2	1	2	1
1824	0	7	0	0	0	2	1	0	2	0
1825	0	28	1	1	1	1	1	0	2	0
1826	0	19	1	0	0	1	1	0	2	0
1827	0	29	1	0	1	1	1	0	2	0
1828	0	29	1	0	1	3	2	0	2	0
1829	0	29	0	0	0	1	1	0	2	0
1830	0	29	1	0	1	1	3	1	2	0
1831	0	29	0	1	0	3	1	0	2	0
1832	0	4	1	0	0	1	1	0	2	0
1833	0	23	0	0	0	3	1	0	2	0
1834	0	28	1	1	1	1	3	1	2	0
1835	0	29	1	1	0	1	2	1	2	0
1836	0	29	1	0	0	2	1	1	2	0
1837	0	29	1	1	1	3	3	1	2	0
1838	0	24	1	1	0	2	2	0	3	0
1839	0	26	0	1	0	2	1	0	1	0
1840	0	29	1	0	1	1	1	0	2	0
1841	1	5	0	0	0	1	2	1	1	1
1842	0	24	0	0	0	3	1	0	2	0
1843	0	29	1	0	0	1	1	0	2	0
1844	0	29	0	0	0	3	1	1	2	0
1845	0	29	0	0	0	1	1	0	2	0
1846	0	28	1	1	0	1	1	0	2	0
1847	0	19	0	0	0	1	1	0	2	0
1848	1	1	0	1	1	1	3	1	3	1
1849	0	29	1	1	0	2	3	1	2	0
1850	0	20	0	1	0	2	2	0	2	0
1851	0	18	1	1	0	1	1	1	2	0
1852	0	25	0	0	0	1	2	1	2	0

1853	0	29	0	0	0	1	2	0	2	0
1854	1	4	1	0	1	1	3	1	2	0
1855	1	5	0	1	0	2	2	1	2	0
1856	0	11	0	0	0	1	1	0	1	0
1857	0	20	0	0	0	1	1	0	1	0
1858	0	29	0	0	0	1	1	0	2	0
1859	0	22	1	1	1	1	2	0	2	0
1860	0	29	1	0	1	1	1	1	2	0
1861	0	22	0	0	0	1	1	0	2	1
1862	0	23	1	1	1	1	1	0	2	0
1863	0	17	0	0	0	1	2	0	3	0
1864	0	12	0	0	0	3	1	0	1	0
1865	0	6	1	0	0	1	1	0	1	0
1866	0	29	0	0	0	2	1	1	2	0
1867	0	29	0	0	0	1	1	0	2	0
1868	0	29	1	1	0	1	2	0	3	0
1869	0	29	1	0	0	2	1	0	2	0
1870	0	4	1	0	0	1	1	0	3	0
1871	0	8	0	0	0	1	1	0	2	0
1872	0	26	1	1	0	1	1	0	1	0
1873	0	29	1	1	0	2	2	1	2	0
1874	0	29	1	1	0	1	1	0	1	0
1875	1	6	0	1	0	1	1	0	1	0
1876	0	29	1	1	1	2	1	0	2	0
1877	0	4	1	1	0	1	1	0	1	0
1878	0	29	0	0	0	1	1	0	1	0
1879	0	29	0	0	0	2	3	0	2	0
1880	0	29	1	1	0	1	1	0	1	0
1881	0	29	0	1	1	2	3	1	2	1
1882	1	5	0	0	0	2	3	0	3	1
1883	1	10	0	1	0	1	1	1	3	0
1884	1	2	0	0	0	1	2	1	2	1
1885	0	27	1	0	0	1	1	0	1	0
1886	0	29	1	1	0	1	1	0	2	0
1887	0	29	1	1	1	2	2	0	2	0
1888	0	2	1	0	1	2	3	1	3	1
1889	0	29	1	0	0	1	2	0	2	0
1890	0	14	1	1	0	1	2	1	2	1

1891	0	13	0	1	0	1	1	1	1	0
1892	0	29	1	1	0	1	3	0	1	0
