# Supplementary material for: Prognostic model of HIV-associated talaromycosis in south China: A large-scale retrospective study
Source: PLoS Negl Trop Dis. 2025 Oct 30;19(10):e0013672. doi: 10.1371/journal.pntd.0013672 (PMC12591474; doi:10.1371/journal.pntd.0013672)
Supplement: S1 Table — (DOCX) [file pntd.0013672.s004.docx]

**S1_Table. Comparation of baseline characteristics between training set, internal validation set and external validation set.**

| **Characters** | **Training set (N=1004)** | **Internal validation set (N=431)** | ***P^1^*-value** | **Independent testing set (N=457)** | ***P^2^*-value** |
| --- | --- | --- | --- | --- | --- |
| Age (yes) | 37.0 [29.8;47.0] | 38.0 [31.0;47.0] | 0.372 | 40.0 [31.0;51.0] | <0.001 |
| Male, n (%) | 798 (79.5%) | 359 (83.3%) | 0.109 | 387 (84.7%) | 0.022 |
| ART experienced, n (%) | 125 (12.5%) | 56 (13.0%) | 0.844 | 104 (22.8%) | <0.001 |
| Fever, n (%) | 806 (80.3%) | 334 (77.5%) | 0.260 | 309 (67.6%) | <0.001 |
| Respiratory systems, n (%) | 697 (69.4%) | 302 (70.1%) | 0.856 | 263 (57.5%) | <0.001 |
| Digestive systems, n (%) | 417 (41.5%) | 176 (40.8%) | 0.851 | 151 (33.0%) | 0.002 |
| Skin lesions, n (%) | 447 (44.5%) | 189 (43.9%) | 0.860 | 174 (38.1%) | 0.024 |
| Lymphadenopathy, n (%) | 732 (72.9%) | 313 (72.6%) | 0.962 | 258 (56.5%) | <0.001 |
| Hepatosplenomegaly, n (%) | 599 (59.7%) | 257 (59.6%) | 1.000 | 228 (49.9%) | 0.001 |
| Breath (/min) | 20.0 [20.0;21.0] | 20.0 [20.0;21.0] | 0.790 | 20.0 [20.0;22.0] | 0.129 |
| Shock index | 0.9 [0.8;1.1] | 0.9 [0.8;1.1] | 0.276 | 0.9 [0.8;1.1] | 0.792 |
| WBC (10^9^) | 4.0 [2.6;5.8] | 4.2 [2.7;5.8] | 0.427 | 4.5 [3.0;6.4] | 0.002 |
| WBC stratifies |  |  | 0.670 |  | 0.027 |
| Normal | 530 (52.8%) | 231 (53.6%) |  | 274 (60.0%) |  |
| Leucopenia | 399 (39.7%) | 163 (37.8%) |  | 149 (32.6%) |  |
| Leucocytosis | 75 (7.5%) | 37 (8.6%) |  | 34 (7.4%) |  |
| Hb (g/L) | 91.3 (21.8) | 91.4 (22.7) | 0.922 | 90.0 [76.0;108.0] | 0.938 |
| Anemia stratifies |  |  | 0.224 |  | 0.248 |
| Normal | 105 (10.5%) | 48 (11.1%) |  | 56 (12.3%) |  |
| Mild (90 g/L -LLN) | 420 (41.8%) | 192 (44.5%) |  | 175 (38.3%) |  |
| Moderate (60 g/L -89 g/L) | 410 (40.8%) | 153 (35.5%) |  | 184 (40.3%) |  |
| Severe (<60 g/L) | 69 (6.9%) | 38 (8.8%) |  | 42 (9.2%) |  |
| PLT (10^9^) | 117.0 [58.8;195.0] | 114.0 [56.5;192.5] | 0.831 | 120.0 [52.0;206.0] | 0.976 |
| Thrombocytopenia stratifies |  |  | 0.728 |  | 0.809 |
| Normal | 562 (56.0%) | 234 (54.3%) |  | 252 (55.1%) |  |
| 30*10^9^-100*10^9^ | 337 (33.6%) | 154 (35.7%) |  | 152 (33.3%) |  |
| <30*10^9^ | 105 (10.5%) | 43 (10.0%) |  | 53 (11.6%) |  |
| TBIL (μmol/L) | 10.0 [7.1;17.5] | 9.9 [7.0;17.1] | 0.619 | 9.0 [6.0;17.0] | 0.011 |
| TBIL elevation | 131 (13.0%) | 58 (13.5%) | 0.901 | 71 (15.5%) | 0.232 |
| ALT (U/L) | 36.0 [22.0;65.0] | 38.0 [23.0;63.0] | 0.710 | 30.0 [16.9;54.0] | <0.001 |
| ALT stratifies |  |  | 0.577 |  | 0.001 |
| <1 ULN | 627 (62.5%) | 275 (63.8%) |  | 329 (72.0%) |  |
| 1-5 ULN | 355 (35.4%) | 150 (34.8%) |  | 118 (25.8%) |  |
| >5 ULN | 22 (2.2%) | 6 (1.4%) |  | 10 (2.2%) |  |
| AST (U/L) | 80.0 [41.0;161.0] | 77.0 [43.0;170.0] | 0.814 | 67.0 [34.0;147.0] | 0.007 |
| AST stratifies |  |  | 0.483 |  | 0.035 |
| <1 ULN | 245 (24.4%) | 99 (23.0%) |  | 141 (30.9%) |  |
| 1-5 ULN | 576 (57.4%) | 242 (56.1%) |  | 240 (52.5%) |  |
| >5 ULN | 183 (18.2%) | 90 (20.9%) |  | 76 (16.6%) |  |
| ALB | 25.0 [21.0;29.0] | 25.0 [21.0;29.0] | 0.707 | 27.0 [23.0;32.0] | <0.001 |
| Severe hypoalbuminemia (<25 g/L) | 465 (46.3%) | 197 (45.7%) | 0.878 | 166 (36.3%) | <0.001 |
| LDH (U/L) | 458.0 [305.8;830.2] | 441.0 [306.0;824.0] | 0.928 | 400.0 [270.0;631.0] | <0.001 |
| LDH stratifies |  |  | 0.810 |  | 0.018 |
| <1 ULN | 141 (14.0%) | 55 (12.8%) |  | 89 (19.5%) |  |
| 1-5 ULN | 726 (72.3%) | 316 (73.3%) |  | 318 (69.6%) |  |
| >5 ULN | 137 (13.6%) | 60 (13.9%) |  | 50 (10.9%) |  |
| AKP (U/L) | 135.5 [83.0;242.2] | 140.0 [90.0;262.5] | 0.118 | 106.0 [75.0;202.0] | <0.001 |
| AKP stratifies |  |  | 0.752 |  | <0.001 |
| <1 ULN | 471 (46.9%) | 196 (45.5%) |  | 268 (58.6%) |  |
| 1-5 ULN | 496 (49.4%) | 216 (50.1%) |  | 179 (39.2%) |  |
| >5 ULN | 37 (3.7%) | 19 (4.4%) |  | 10 (2.2%) |  |
| BUN (mmol/L) | 4.4 [3.2;6.2] | 4.4 [3.3;6.0] | 0.876 | 4.7 [3.5;7.1] | <0.001 |
| BUN elevation | 101 (10.1%) | 47 (10.9%) | 0.698 | 76 (16.6%) | <0.001 |
| UA (μmol/L) | 231.5 [173.0;303.0] | 236.0 [171.2;316.0] | 0.390 | 252.0 [197.0;332.0] | <0.001 |
| UA elevation | 83 (8.3%) | 36 (8.4%) | 1.000 | 65 (14.2%) | 0.001 |
| Cr (μmol/L) | 68.0 [56.0;84.0] | 71.0 [59.0;86.5] | 0.026 | 68.0 [56.0;85.0] | 0.569 |
| Cr elevation: | 106 (10.6%) | 52 (12.1%) | 0.457 | 54 (11.8%) | 0.533 |
| CD4 (cells/μL) | 11.0 [5.0;21.0] | 9.0 [5.0;21.0] | 0.674 | 14.0 [7.0;33.0] | <0.001 |
| CD8 (cells/μL) | 233.0 [127.0;392.2] | 224.0 [123.0;389.5] | 0.647 | 233.0 [117.0;418.0] | 0.601 |
| CD4/CD8 ratio | 0.05 [0.03;0.09] | 0.05 [0.03;0.08] | 0.851 | 0.07 [0.03;0.13] | <0.001 |

Categorical variables were represented as n (%), whereas continuous variables were described using either the median and interquartile range (IQR) or the mean and standard deviation, contingent upon the data distribution.

ART: antiretroviral therapy; n (%): number (percentage); IQR: interquartile range; WBC: white blood cell; Hb: hemoglobin; LLN: lower limit of normal; PLT: platelet; TBIL: total bilirubin; ALT: alanine aminotransferase; ULN: upper limit of normal; AST: aspartate aminotransferase; ALB: albumin; LDH: lactate dehydrogenase; AKP: alkaline phosphatase; BUN: blood urea nitrogen; UA: uric acid; Cr: creatinine.
