# Supplementary material for: Prognostic model of HIV-associated talaromycosis in south China: A large-scale retrospective study
Source: PLoS Negl Trop Dis. 2025 Oct 30;19(10):e0013672. doi: 10.1371/journal.pntd.0013672 (PMC12591474; doi:10.1371/journal.pntd.0013672)
Supplement: S3 Table — (DOCX) [file pntd.0013672.s006.docx]

**S3_Table. Comparation of baseline characteristics between patients admitted at winter and non-winter.**

| **Characters** | **Total**  **(N=1892)** | **Seasons** | |  |
| --- | --- | --- | --- | --- |
|  |  | **Winer**  **(N=360)** | **Non-winter**  **(N=1532)** | ***P*-value** |
| Age (yes) | 38.0 [30.0;48.0] | 39.0 [30.0;48.0] | 38.0 [30.0;48.0] | 0.993 |
| Male, n (%) | 1544 (81.6%) | 295 (81.9%) | 1249 (81.5%) | 0.914 |
| ART experienced, n (%) | 285 (15.1%) | 71 (19.7%) | 214 (14.0%) | 0.008 |
| Fever, n (%) | 1449 (76.6%) | 254 (70.6%) | 1195 (78.0%) | 0.003 |
| Respiratory systems, n (%) | 1262 (66.7%) | 229 (63.6%) | 1033 (67.4%) | 0.187 |
| Digestive systems, n (%) | 744 (39.3%) | 139 (38.6%) | 605 (39.5%) | 0.804 |
| Skin lesions, n (%) | 810 (42.8%) | 156 (43.3%) | 654 (42.7%) | 0.870 |
| Lymphadenopathy, n (%) | 1303 (68.9%) | 226 (62.8%) | 1077 (70.3%) | 0.007 |
| Hepatosplenomegaly, n (%) | 1084 (57.3%) | 200 (55.6%) | 884 (57.7%) | 0.495 |
| Breath rate (/min) | 20.0 [20.0;21.0] | 20.0 [20.0;22.0] | 20.0 [20.0;21.0] | 0.012 |
| Tachypnea | 436 (23.0%) | 99 (27.5%) | 337 (22.0%) | 0.031 |
| Shock index | 0.9 [0.8;1.1] | 0.9 [0.8;1.1] | 0.9 [0.8;1.1] | 0.262 |
| WBC (10^9^) | 4.1 [2.8;6.0] | 4.2 [2.7;6.1] | 4.1 [2.8;6.0] | 0.947 |
| WBC stratifies |  |  |  | 0.641 |
| Normal | 1035 (54.7%) | 190 (52.8%) | 845 (55.2%) |  |
| Leucopenia | 711 (37.6%) | 139 (38.6%) | 572 (37.3%) |  |
| Leucocytosis | 146 (7.7%) | 31 (8.6%) | 115 (7.5%) |  |
| Hb (g/L) | 91.4 (22.3) | 90.3 (22.4) | 91.6 (22.3) | 0.313 |
| Anemia stratifies |  |  |  | 0.539 |
| Normal | 209 (11.0%) | 34 (9.4%) | 175 (11.4%) |  |
| Mild (90 g/L -LLN) | 787 (41.6%) | 147 (40.8%) | 640 (41.8%) |  |
| Moderate (60 g/L -89 g/L) | 747 (39.5%) | 146 (40.6%) | 601 (39.2%) |  |
| Severe (<60 g/L) | 149 (7.9%) | 33 (9.2%) | 116 (7.6%) |  |
| PLT (10^9^) | 117.5 [56.0;197.0] | 106.5 [56.0;195.0] | 119.0 [56.0;198.2] | 0.499 |
| Thrombocytopenia stratifies |  |  |  | 0.233 |
| Normal | 1048 (55.4%) | 191 (53.1%) | 857 (55.9%) |  |
| 30*10^9^-100*10^9^ | 643 (34.0%) | 122 (33.9%) | 521 (34.0%) |  |
| <30*10^9^ | 201 (10.6%) | 47 (13.1%) | 154 (10.1%) |  |
| TBIL (μmol/L) | 9.9 [7.0;17.3] | 9.0 [6.7;16.7] | 10.0 [7.0;17.5] | 0.294 |
| TBIL elevation | 260 (13.7%) | 50 (13.9%) | 210 (13.7%) | 0.996 |
| ALT (U/L) | 35.0 [21.0;62.0] | 36.0 [20.0;61.0] | 35.0 [21.0;62.0] | 0.773 |
| ALT stratifies |  |  |  | 0.952 |
| <1 ULN | 1231 (65.1%) | 232 (64.4%) | 999 (65.2%) |  |
| 1-5 ULN | 623 (32.9%) | 121 (33.6%) | 502 (32.8%) |  |
| >5 ULN | 38 (2.0%) | 7 (1.9%) | 31 (2.0%) |  |
| AST (U/L) | 76.0 [39.0;159.2] | 74.0 [37.0;162.8] | 77.0 [39.0;158.2] | 0.900 |
| AST stratifies |  |  |  | 0.664 |
| <1 ULN | 485 (25.6%) | 95 (26.4%) | 390 (25.5%) |  |
| 1-5 ULN | 1058 (55.9%) | 194 (53.9%) | 864 (56.4%) |  |
| >5 ULN | 349 (18.4%) | 71 (19.7%) | 278 (18.1%) |  |
| ALB | 25.0 [22.0;29.0] | 25.0 [21.0;29.0] | 25.0 [22.0;29.0] | 0.040 |
| Severe hypoalbuminemia | 828 (43.8%) | 174 (48.3%) | 654 (42.7%) | 0.060 |
| LDH (U/L) | 437.0 [295.0;776.0] | 419.5 [280.2;716.0] | 440.0 [297.0;801.2] | 0.110 |
| LDH stratifies |  |  |  | 0.050 |
| <1 ULN | 285 (15.1%) | 69 (19.2%) | 216 (14.1%) |  |
| 1-5 ULN | 1360 (71.9%) | 244 (67.8%) | 1116 (72.8%) |  |
| >5 ULN | 247 (13.1%) | 47 (13.1%) | 200 (13.1%) |  |
| AKP (U/L) | 128.0 [82.0;237.0] | 124.5 [79.8;250.8] | 128.5 [82.0;231.0] | 0.758 |
| AKP stratifies |  |  |  | 0.187 |
| <1 ULN | 935 (49.4%) | 180 (50.0%) | 755 (49.3%) |  |
| 1-5 ULN | 891 (47.1%) | 162 (45.0%) | 729 (47.6%) |  |
| >5 ULN | 66 (3.5%) | 18 (5.0%) | 48 (3.1%) |  |
| BUN (mmol/L) | 4.5 [3.3;6.3] | 4.7 [3.5;7.1] | 4.4 [3.3;6.1] | 0.006 |
| BUN elevation | 224 (11.8%) | 65 (18.1%) | 159 (10.4%) | <0.001 |
| UA (μmol/L) | 238.2 [177.5;315.0] | 234.3 [174.7;323.5] | 238.6 [178.0;314.0] | 0.908 |
| UA elevation | 184 (9.7%) | 43 (11.9%) | 141 (9.2%) | 0.139 |
| Cr (μmol/L) | 68.1 [57.0;85.0] | 68.3 [56.8;86.0] | 68.0 [57.0;85.0] | 0.698 |
| Cr elevation: | 212 (11.2%) | 50 (13.9%) | 162 (10.6%) | 0.089 |
| CD4 (cells/μL) | 11.0 [5.0;23.0] | 10.0 [5.0;22.2] | 12.0 [5.0;23.0] | 0.345 |
| CD8 (cells/μL) | 233.0 [123.0;395.2] | 223.0 [113.2;374.8] | 235.0 [126.0;401.5] | 0.234 |
| CD4_CD8_ratio | 0.050 [0.030;0.090] | 0.050 [0.030;0.100] | 0.050 [0.030;0.090] | 0.867 |

Categorical variables were represented as n (%), whereas continuous variables were described using either the median and interquartile range (IQR) or the mean and standard deviation, contingent upon the data distribution.

ART: antiretroviral therapy; n (%): number (percentage); IQR: interquartile range; WBC: white blood cell; Hb: hemoglobin; LLN: lower limit of normal; PLT: platelet; TBIL: total bilirubin; ALT: alanine aminotransferase; ULN: upper limit of normal; AST: aspartate aminotransferase; ALB: albumin; LDH: lactate dehydrogenase; AKP: alkaline phosphatase; BUN: blood urea nitrogen; UA: uric acid; Cr: creatinine.
