# Supplementary material for: Prognostic model of HIV-associated talaromycosis in south China: A large-scale retrospective study
Source: PLoS Negl Trop Dis. 2025 Oct 30;19(10):e0013672. doi: 10.1371/journal.pntd.0013672 (PMC12591474; doi:10.1371/journal.pntd.0013672)
Supplement: S4 Table — (DOCX) [file pntd.0013672.s007.docx]

**S4_Table. Antifungal therapy-adjusted multivariate analysis​.**

| **Characters** | **Univariate analysis** |  | **Multivariate analysis** |  |
| --- | --- | --- | --- | --- |
|  | **HR (95%CI)** | ***P* value** | **aHR (95%CI)** | ***P* value** |
| AMB | 0.323 (0.222-0.472) | <0.001 | 0.404 (0.272-0.599) | <0.001 |
| Age (yes) | 1.008 (0.994-1.023) | 0.267 |  |  |
| Male | 0.938 (0.994-1.023) | 0.774 |  |  |
| Season |  |  |  |  |
| Spring | Ref (1.00) |  |  |  |
| Summer | 0.962 (0.994-1.023) | 0.876 |  |  |
| Autumn | 0.944 (0.994-1.023) | 0.834 |  |  |
| Winter | 1.620 (0.994-1.023) | 0.043 |  |  |
| ART experienced | 1.006 (0.994-1.023) | 0.983 |  |  |
| Fever | 1.158 (0.994-1.023) | 0.542 |  |  |
| Respiratory systems | 1.072 (0.994-1.023) | 0.731 |  |  |
| Digestive systems | 1.651 (0.994-1.023) | 0.006 |  |  |
| Skin lesions | 1.102 (0.994-1.023) | 0.597 |  |  |
| Lymphadenopathy | 0.403 (0.994-1.023) | <0.001 | 0.592 (0.402-0.871) | 0.008 |
| Hepatosplenomegaly | 0.389 (0.269-0.563) | <0.001 | 0.359 (0.239-0.539) | <0.001 |
| Breath (/min) | 1.139 (1.109-1.170) | <0.001 | 1.047 (1.014-1.081) | 0.005 |
| Shock index | 4.046 (1.880-8.704) | <0.001 |  |  |
| WBC (10^9^) | 1.144 (1.108-1.181) | <0.001 | 1.097 (1.058-1.137) | <0.001 |
| Hb (g/L) | 0.989 (0.981-0.998) | 0.014 | 0.993 (0.984-1.002) | 0.116 |
| PLT (10^9^) | 0.992 (0.990-0.995) | <0.001 | 0.995 (0.993-0.998) | <0.001 |
| TBIL (μmol/L) | 1.007 (1.003-1.011) | <0.001 |  |  |
| ALT (U/L) | 1.001 (1.001-1.002) | <0.001 |  |  |
| AST (U/L) | 1.001 (1.001-1.001) | <0.001 |  |  |
| ALB (g/L) | 0.852 (0.818-0.887) | <0.001 | 0.920 (0.879-0.962) | <0.001 |
| LDH (U/L) | 1.000 (1.000-1.000) | <0.001 | 1.000 (1.000-1.000) | <0.001 |
| AKP (U/L) | 1.001 (1.000-1.002) | 0.001 |  |  |
| BUN (mmol/L) | 1.117 (1.101-1.134) | <0.001 | 1.075 (1.055-1.095) | <0.001 |
| UA (μmol/L) | 1.003 (1.003-1.004) | <0.001 |  |  |
| Cr (μmol/L) | 1.004 (1.003-1.005) | <0.001 |  |  |
| CD4 (cells/μL) | 1.000 (0.997-1.003) | 0.807 |  |  |
| CD8 (cells/μL) | 1.000 (0.999-1.000) | 0.401 |  |  |

Multivariate Cox regression models, backward Wald method was used.

AMB: amphotericin B; ART: antiretroviral therapy; WBC: white blood cell; Hb: hemoglobin; PLT: platelet; TBIL: total bilirubin; ALT: alanine aminotransferase; AST: aspartate aminotransferase; ALB: albumin; LDH: lactate dehydrogenase; AKP: alkaline phosphatase; BUN: blood urea nitrogen; UA: uric acid; Cr: creatinine; HR: hazard ratio; CI: confidence interval; Ref: reference; aHR: adjusted hazard ratio.
