# Supplementary figures and images for: Prognostic model of HIV-associated talaromycosis in south China: A large-scale retrospective study
Source: PLoS Negl Trop Dis. 2025 Oct 30;19(10):e0013672. doi: 10.1371/journal.pntd.0013672 (PMC12591474; doi:10.1371/journal.pntd.0013672)

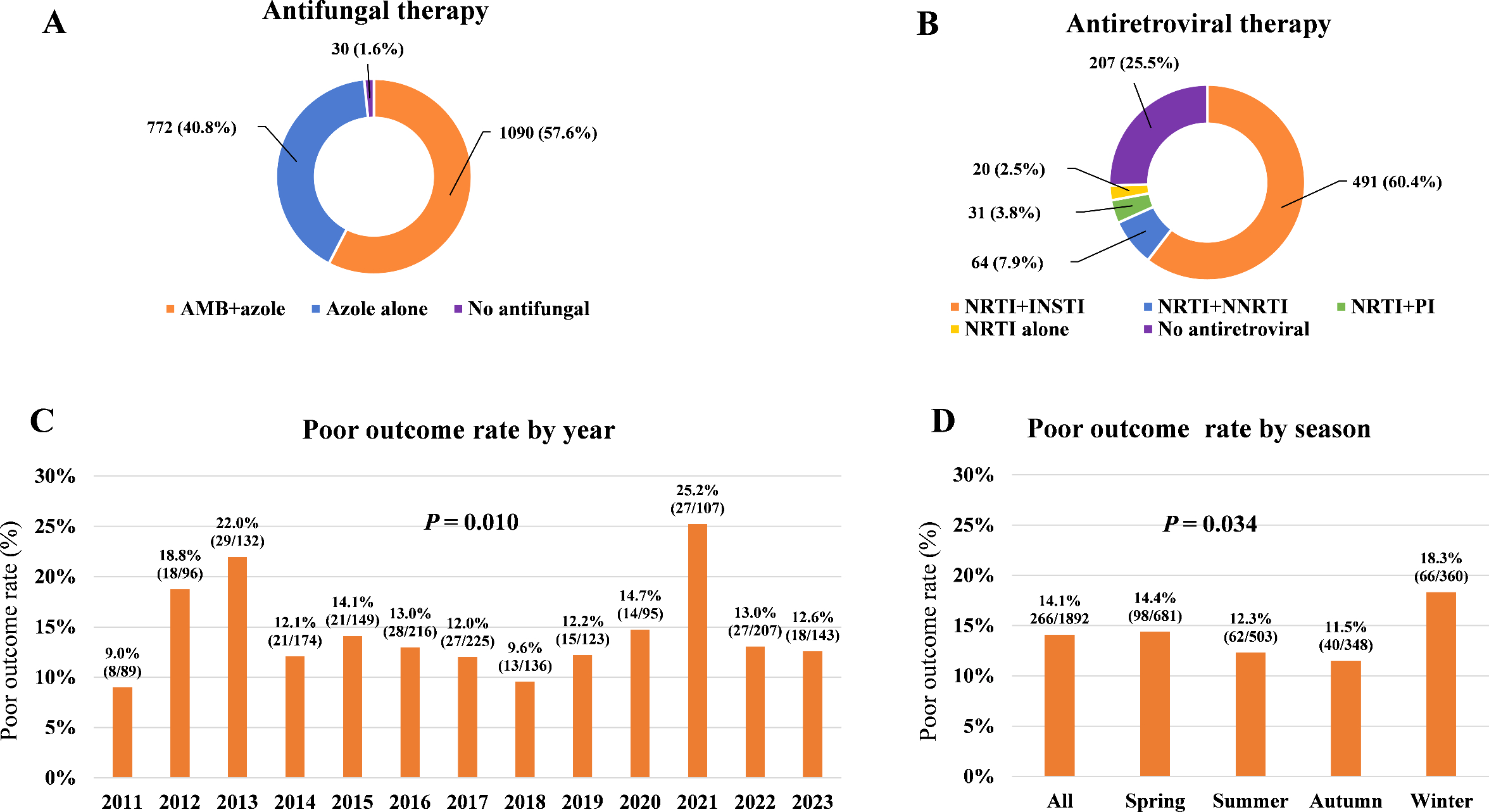

Supplement: S1 Fig — (A) Antifungal therapy distribution: amphotericin B (AMB)-based regimens (57.6%), azole monotherapy (40.8%), no therapy (1.6%); (B) Antiretroviral therapy distribution for 813 patients who admitted between January 1, 2018 and December 31, 2023; (C) Interannual poor outcome rates (2011–2023, P = 0.010); (D) Seasonal incidence and poor outcome rates (winter: 18.3% vs. summer: 12.3%, autumn: 11.5%; P = 0.034). (TIF) [file pntd.0013672.s008.tif]

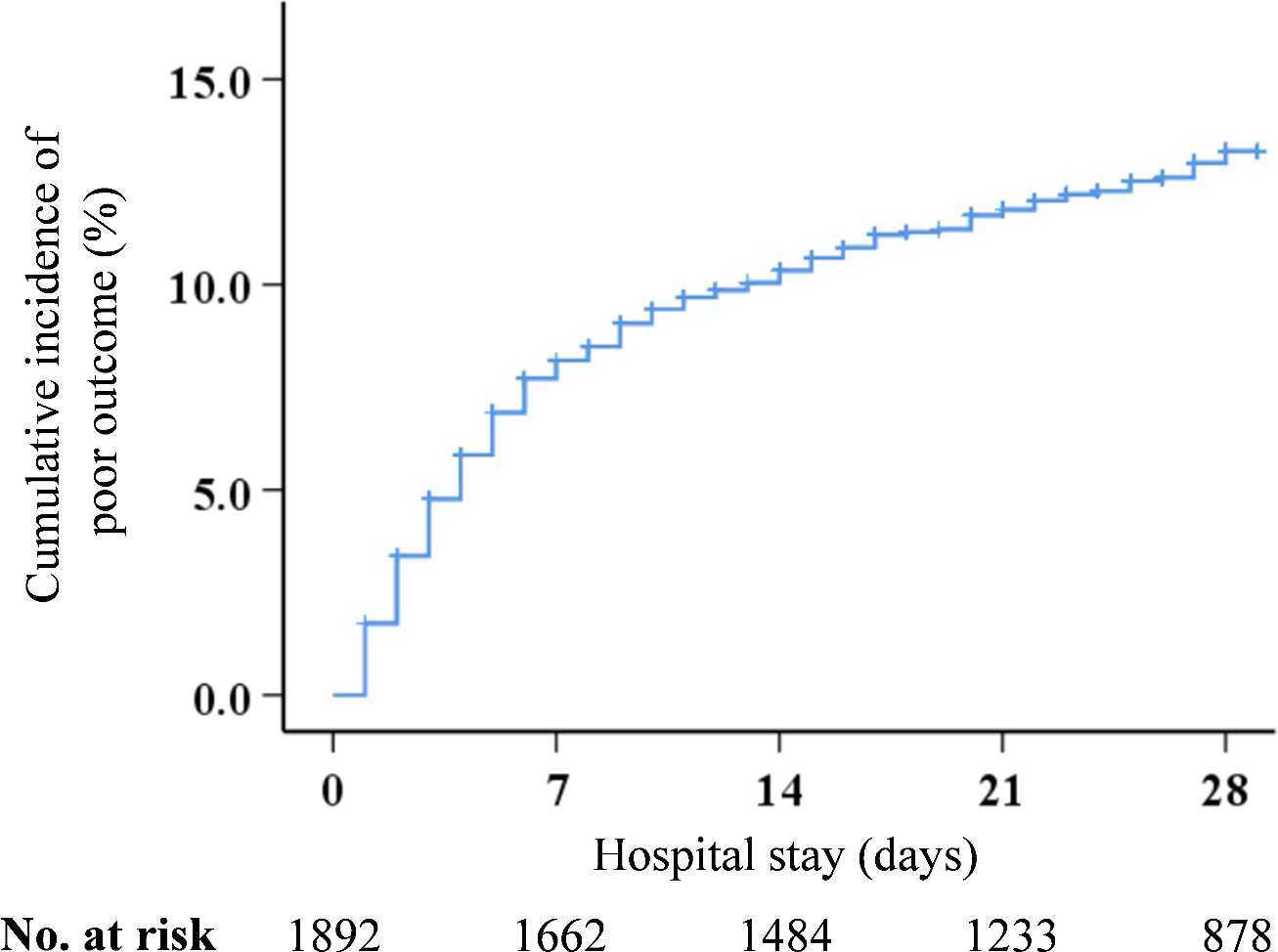

Supplement: S2 Fig — 86.5% (230/266) of poor outcomes occurred within 28 days (7-day: 8.2%, 14-day: 10.4%, 28-day: 13.4%). (TIF) [file pntd.0013672.s009.tif]

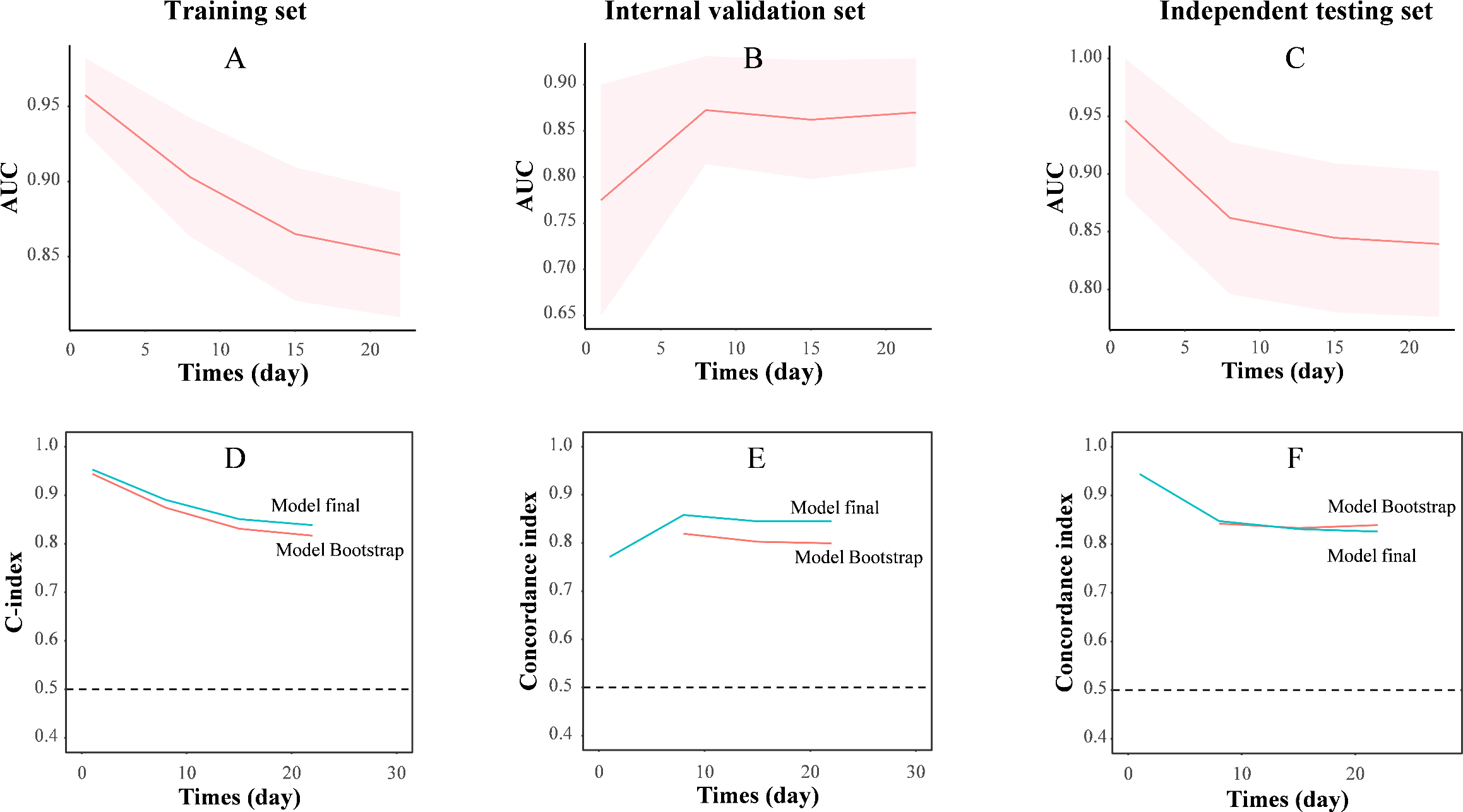

Supplement: S3 Fig — (A-C) Time-dependent area under the curve values for 28-day prediction; (D-F) Concordance index stability over time in (A, D) training, (B, E) internal validation, and (C, F) independent testing sets. Gray band indicates 500 rounds of bootstrap confidence intervals. (TIF) [file pntd.0013672.s010.tif]

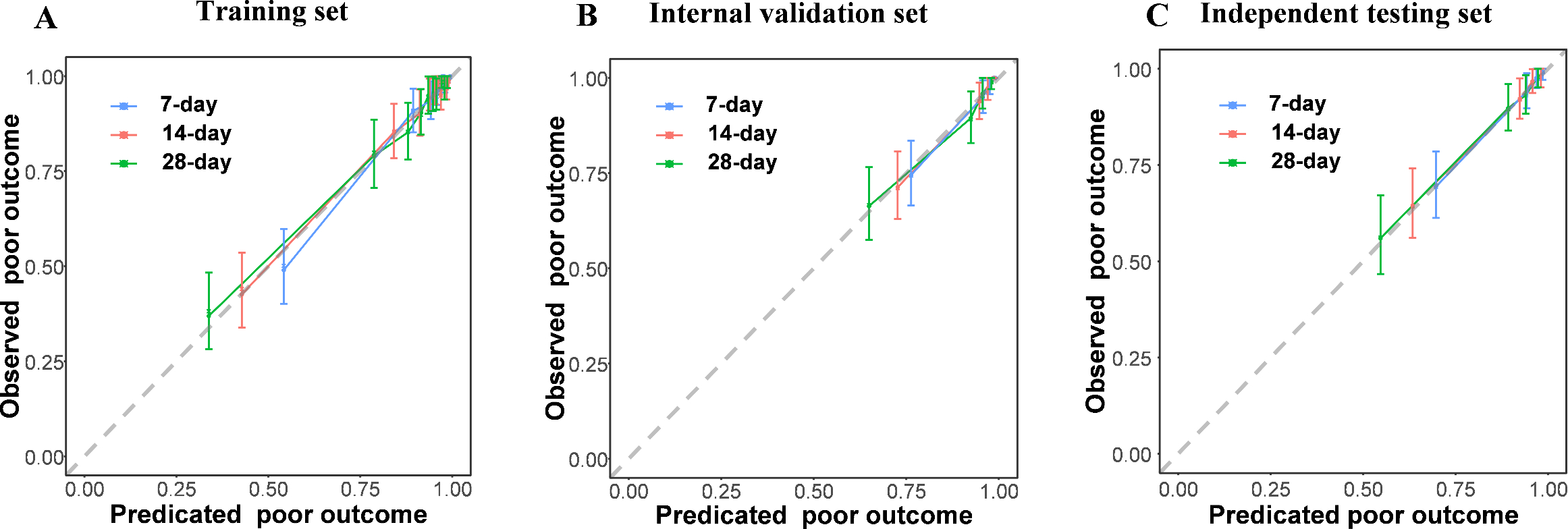

Supplement: S4 Fig — (A-C) Agreement between predicted and observed poor outcome probabilities at 7, 14, and 28 days in (A) training, (B) internal validation, and (C) independent testing sets. Dashed line represents perfect calibration. (TIF) [file pntd.0013672.s011.tif]

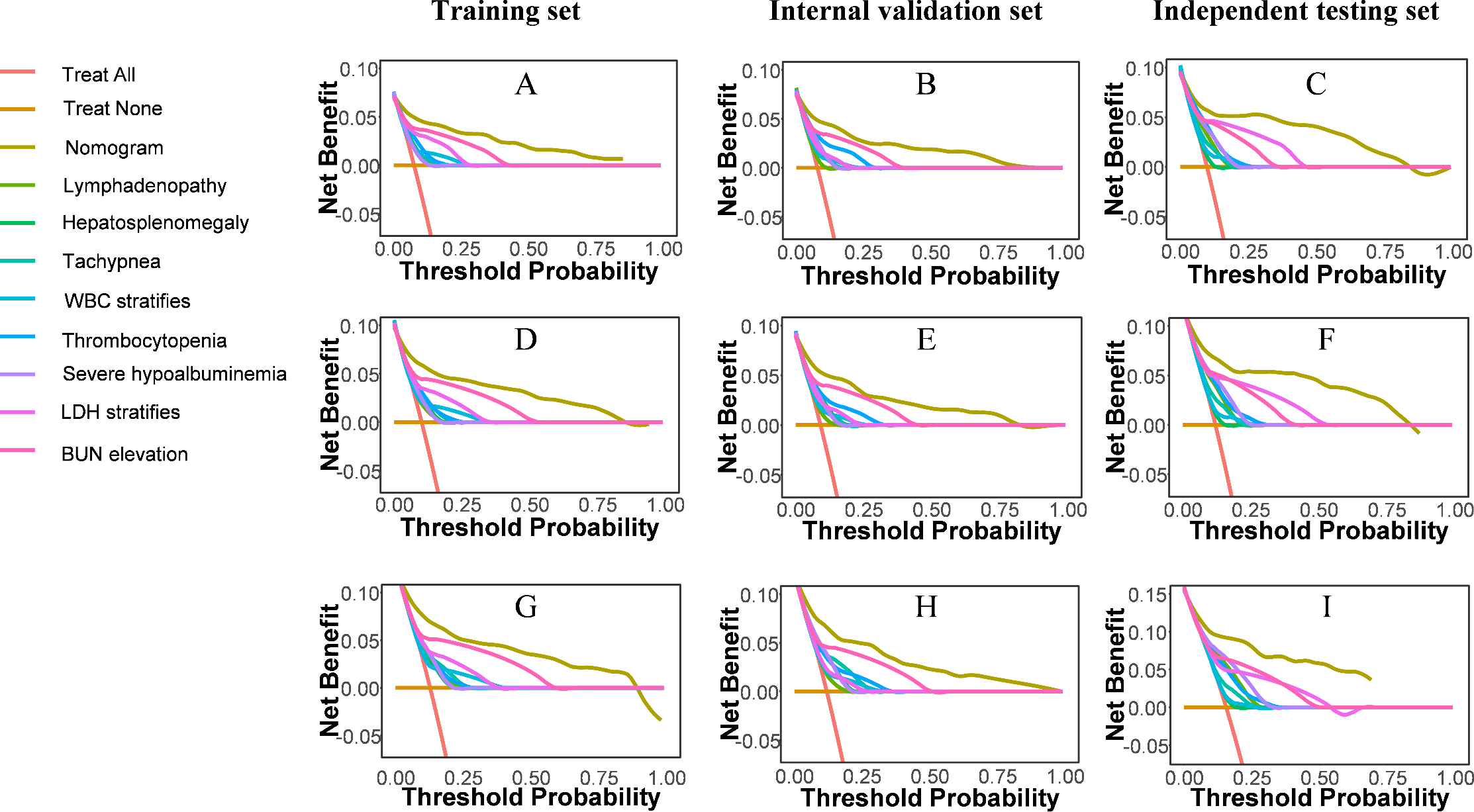

Supplement: S5 Fig — (A-I) Net benefit of the nomogram compared to “treat all” or “treat none” strategies across threshold probabilities (0–100%) for: (A, D, G) training set; (B, E, H) internal validation; (C, F, I) independent testing. (TIF) [file pntd.0013672.s012.tif]

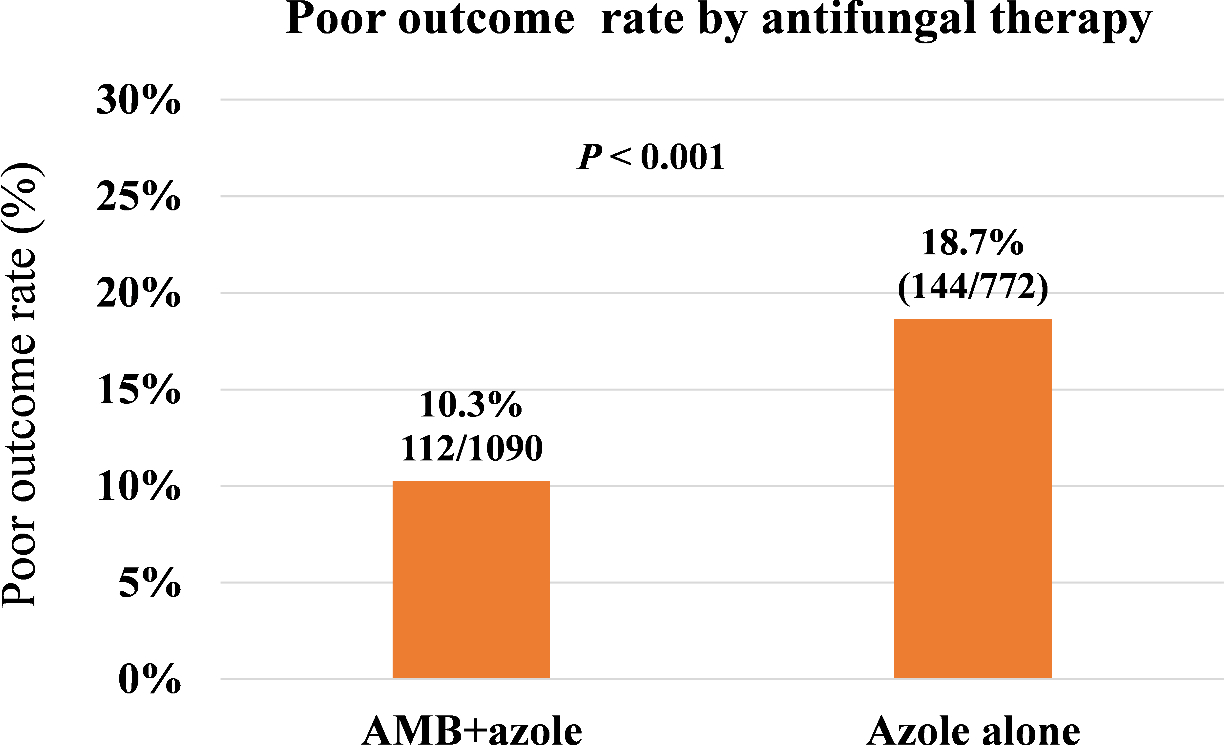

Supplement: S6 Fig — Bar plot demonstrating significantly lower incidence of poor outcomes in patients receiving amphotericin B-azole combination therapy (10.3%, 112/1090) compared to azole monotherapy (18.7%, 144/772) (P < 0.001). (TIF) [file pntd.0013672.s013.tif]
